# Antagonistic Ghd7‐OsNAC42 Complexes Modulate Carbon and Nitrogen Metabolism to Achieves Superior Quality and High Yield in Rice

**DOI:** 10.1002/advs.202504163

**Published:** 2025-06-10

**Authors:** Guangming Lou, Pingli Chen, Pingbo Li, Haozhou Gao, Jiawang Xiong, Shanshan Wan, Yuanyuan Zheng, Yufu Wang, Mufid Alam, Yingnanjun Chen, Lei Wang, Jingjing Bai, Xuan Tan, Wenting Rao, Bian Wu, Hao Zhou, Yanhua Li, Guanjun Gao, Qinglu Zhang, Jinghua Xiao, Xianghua Li, Xuelei lai, Qifa Zhang, Yuqing He

**Affiliations:** ^1^ National Key Laboratory of Crop Genetic Improvement and National Center of Plant Gene Research (Wuhan) Hubei Hongshan Laboratory Huazhong Agricultural University Wuhan 430070 China

**Keywords:** carbon and nitrogen metabolism, Ghd7, grain quality, OsNAC42, protein content, rice

## Abstract

Close coordination of carbon and nitrogen (C/N) metabolism is necessary to maintain optimal growth and development of plants and other cellular organisms. The central regulator for achieving high‐yield and quality synergy in rice through regulating the dynamic C/N balance has seldom been reported. Here, the novel function of *Grain number, plant height, and heading date7 (Ghd7)* gene are reported as both a negative regulator of grain protein content and a positive regulator of rice grain quality (including appearance quality and eating quality). As a transcription factor with both activating and inhibitory functions, Ghd7 directly binds to CCACC motif genes involved in C/N metabolism. OsNAC42, an interacting protein of Ghd7, antagonistically regulates the expression of these target genes in rice seedlings and endosperm by forming a heterodimer with Ghd7. The antagonistic Ghd7‐OsNAC42 module can flexibly regulate C/N metabolism in vivo in response to various nitrogen levels, thereby maintaining a dynamic C/N balance. Phenotypically, OsNAC42 enhances grain protein content but compromises quality‐functions opposing Ghd7's effects. In summary, the discovery of the Ghd7‐OsNAC42 antagonistic module provides new insights into the synergy between superior quality and high yield in rice.

## Graphical abstract

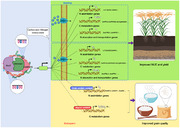

## 1 Introduction

Current knowledge suggests that plants have evolved a complex set of sensing and signaling mechanisms to robustly monitor and appropriately respond to dynamic changes in their surrounding environment.^[^ ^1^ ^]^ The ability to sense carbon and nitrogen metabolites allows plants to regulate metabolism and development according to their internal C to N ratio. For example, the C:N sensing mechanism enables plants to switch off nitrate uptake and reduction when reduced N or organic N is high, while activating genes involved in nitrogen assimilation when the carbon skeleton is abundant and internal organic N levels are low.^[^ ^2^ ^]^ C and N signals are used to coordinate the metabolism and transport of these metabolites between source and sink tissues throughout the life cycle and can also serve as metabolic signals integrating environmental inputs (e.g., light) with internal regulators (e.g., hormones). The sensing and regulation mechanism of this carbon and nitrogen signal also significantly affects the important agronomic traits (yield and quality) of human concern. Therefore, it is particularly important to discover and identify transcription factors that sense and transduce this carbon‐nitrogen signal, and to clarify its impact on crop yield and quality.

Rice (*Oryza sativa* L.) is a staple food that feeds more than half of the world population.^[^ ^3^ ^]^ Polished rice grain consists of two major components, starch and protein, which respectively account for about 90% and 5%–12% of grain dry weight and jointly determine a large part of grain quality, including appearance quality, cooking and eating quality, nutritional quality, and processing quality.^[^ ^4^ ^]^ Superior cooking and eating quality or palatability is being increasingly demanded by consumers,^[^ ^5^ ^]^ and quality improvement has always been an important goal in breeding.^[^ ^6^ ^]^ Thus, identifying genes conferring starch or protein content and evaluating their effects on quality is of considerable importance. Starch has long been studied as the most important factor affecting cooking and eating quality,^[^ ^7^, ^8^, ^9^, ^10^, ^11^ ^]^ but protein has not received enough attention. Although some genes contributing to protein content have been identified from mutants,^[^ ^12^, ^13^, ^14^, ^15^, ^16^, ^17^ ^]^ only *OsAAP6* ^[^ ^18^ ^]^ and *Os* *GluA2* ^[^ ^19^ ^]^ that display natural variation among cultivars have been cloned. Both are positive regulators of grain protein content. More natural variants affecting grain protein content need to be discovered and evaluated for their effects on quality in order to breed for superior quality. The widespread use of semi‐dwarf varieties in cereals since the 1960s has immensely boosted crop yields by reducing lodging under high‐nitrogen fertilizer inputs.^[^ ^20^, ^21^ ^]^ To achieve high productivity in crops, a large amount of nitrogen fertilizer is used by farmers. However, only 30%–40% of the applied nitrogen is absorbed by plants,^[^ ^22^ ^]^ with the remaining released into the environment and causing environmental problems, such as soil hardening and acidification, and eutrophication.^[^ ^23^, ^24^ ^]^ High nitrogen levels increase grain protein content, and high protein content is negatively correlated with the palatability of cooked rice.^[^ ^25^ ^]^ Therefore, development of rice varieties with high nitrogen‐use efficiency (NUE) could produce high grain yield at low nitrogen input, improve cooking and eating quality, and relieve environmental pressure, thus contributing to sustainable agricultural practices.

NUE refers to the plant dry matter that contains per unit mass of nitrogen resulting from the coordination of plant carbon and nitrogen metabolism during growth.^[^ ^26^ ^]^ Maintaining the coordination of carbon and nitrogen metabolism and an appropriate balance between carbohydrates and nitrogen metabolites is also known as the “C/N balance”, which is generally considered to be essential for plant development and determination of yield.^[^ ^26^ ^]^ For starch and protein synthesis during rice grain filling, nitrogen metabolism provides ADPG pyrophosphorylase (ADPG‐PPase), granule‐bound starch synthase (GBSS), soluble starch synthase (SSS), starch branching enzyme (SBE), and starch debranching enzyme (DBE) for carbon metabolism (starch synthesis).^[^ ^27^ ^]^ Carbon metabolism processes such as glycolysis, the pentose phosphate pathway, and tricarboxylic acid cycle provide the necessary carbon skeleton for nitrogen metabolism (amino acid synthesis) (3‐phosphoglyceric acid, pyruvate and phosphoenolpyruvate, red moss 4‐phosphate, 5‐phosphate ribose, oxaloacetic acid, and α‐ketoglutaric acid).^[^ ^26^ ^]^ The final effect of carbon‐nitrogen metabolism determines the quantity and storage state of starch and protein in endosperm, as well as the ratio of amylopectin to amylose, which further affects the yield and quality of rice. In recent years, more and more studies have shown that C/N balance may be the key point of coordinated regulation of yield and quality. By regulating carbon and nitrogen metabolism, the genes delay senescence and increase yield.^[^ ^28^ ^]^ *GRF4* is a positive regulator of plant carbon‐nitrogen metabolism, promoting nitrogen absorption, assimilation, and transport, as well as photosynthesis and carbohydrate metabolism and transport, thereby promoting growth and development.^[^ ^20^ ^]^ *DEP1* is involved in regulating the carbon‐nitrogen metabolic balance to affect grain yield and quality.^[^ ^29^ ^]^ *FLO12* encoding alanine aminotransferase 1 affects grain appearance quality and yield through regulating carbon and nitrogen metabolism.^[^ ^30^ ^]^ Mutation of *FLO19* disrupts the balance of carbon and nitrogen metabolism, resulting in changes to the quality of phenotypes and reduced yield.^[^ ^31^ ^]^ The tiller number, seed setting rate, and thousand‐grain weight of the *abc1* mutant were significantly reduced, while the grain protein content increased significantly and showed a chalky phenotype, indicating that *ABC1* is also a key regulatory factor for carbon and nitrogen balance, which is crucial for plant growth and development.^[^ ^32^ ^]^ Therefore, a coordinated balance of carbon and nitrogen metabolism provides the physiological and biochemical framework for achieving high‐quality and high‐yield.

In conclusion, the contradiction between crop quality and yield, and how to improve nitrogen use efficiency, ultimately lies in the regulation of C/N balance. The desired state of C/N metabolism of human beings must be that nutrient elements are fully utilized, and the photosynthetic products are used as much as possible to synthesize the parts (seeds or fruits) needed by human beings, while the quality can be guaranteed. Therefore, it is of great theoretical significance to explore as many gene resources as possible that can synergistically improve crop yield and quality, and to crack the regulation mechanism of crops to maintain their own C/N balance for the cultivation of new varieties with high quality and high yield.

In this study, we found that *Ghd7* is a gene that can regulate the C/N balance of rice seedlings and grains and ultimately synergistically improve the rice yield and quality. Ghd7 is a negative regulator of grain protein content, and participates in the regulation of multiple transcription pathways and physiological and biochemical metabolic processes as a transcription activator assisted by OsNAC42. In this process, OsNAC42 and Ghd7 formed heterodimer complexes that antagonized each other to jointly regulate the expression of target genes related to C/N metabolism in seedlings and endosperm, thereby maintaining the homeostasis of C/N metabolism in rice. Overall, the antagonism of the Ghd7‐OsNAC42 module is the key for Ghd7 to synergistically improve rice quality and yield.

## 2 Results

### 2.1 *Ghd7* is a Negative Regulator of Grain Protein Content

**Figure 1:**
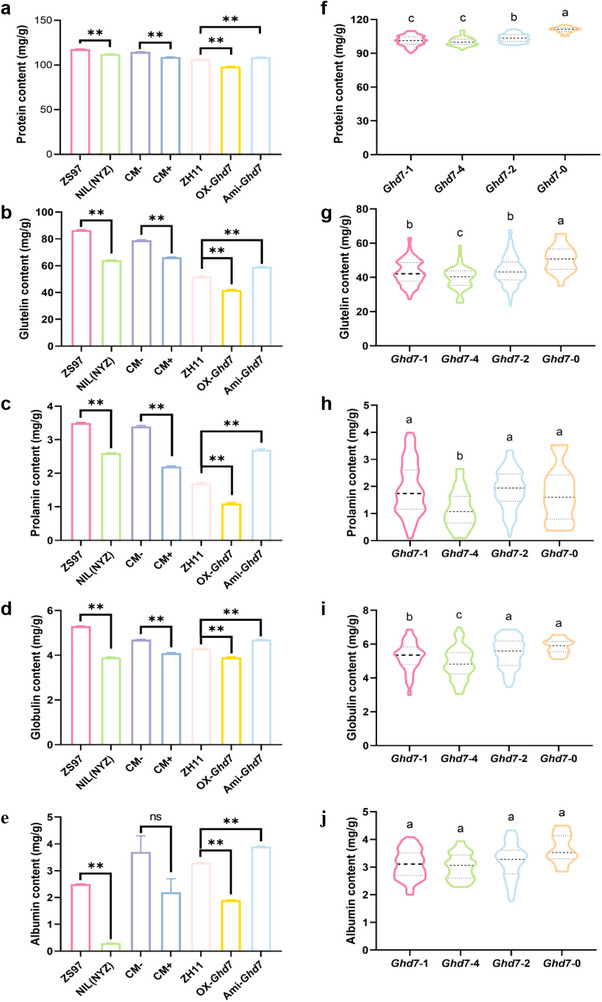
*Ghd7* negatively regulates grain protein content. Contents of total protein and its four main storage components in grains of a‐e) NILs and transgenic lines, *n* ≥ 16; f‐j) the four most frequent haplotypes. Data are means ± s.e.m (*Ghd7*‐1, *n* = 202; *Ghd7*‐4, *n* = 73; *Ghd7‐2*, *n* = 177; *Ghd7*‐0, *n* = 26). Different letters represent significant differences at *p* < 0.05, Duncan's multiple range test. In (a‐e), ** indicates a significant difference at *p *< 0.01, and ns indicates no significant difference, *t*‐tests.

Wang et al.^[^ ^33^ ^]^ identified a quantitative trait locus (QTL) that affected amino acid contents in the grain and the allele from an *Xian*/*Indica* (*XI*)^[^ ^34^, ^35^ ^]^ rice accession Zhenshan 97 (ZS97) increased the contents of all 17 amino acids assayed (including Asp, Thr, Ser, Glu, Gly, Ala, Cys, Val, Met, Ile, Leu, Tyr, Phe, Lys, His, Arg, Pro). Since amino acids make up protein, the QTL was named *qPC7* (for grain protein content on chromosome 7). We chose grain protein content rather than individual amino acids as the target trait in this study. A near‐isogenic line (NIL) named as NIL(NYZ) was developed by introgressing the *qPC7* allele from variety Nanyangzhan (NYZ) into ZS97.^[^ ^36^ ^]^ ZS97 showed a higher grain protein content but lower plant height than NIL(NYZ) (**Figure** **1**a, Figure S1a, Supporting Information), paralleling the difference between ZS97 and NYZ (Figure S1b,c, Supporting Information). In the BC_3_F_2_ population the ratio of plants with low grain protein content to high grain protein content was 3:1 (*n *= 148, χ^2^ = 0.108 <χ^2^_0.05, 1_ = 3.84, *P* > 0.05), indicating the presence of a single dominant allele for low grain protein content from NYZ (Table S1, Supplementary Table). Positional cloning identified *qPC7* as *Ghd7* (the *Ghd7* allele in NYZ was earlier named *Ghd7*‐2), suggesting a novel function of *Ghd7* in the regulation of grain protein content (Figure S2a–c and Note S1, Supporting Information).

Notably, since distinct heading date results in distinct terms for maturing seeds under distinct environmental conditions in fields, some scholars maintain that the differences in rice quality are primarily attributed to the significant influence of *Ghd7* on heading date. To further investigate the extent to which the environment may affect the impact of *Ghd7* on grain protein content, we examined ZS97 and NIL(NYZ) across two plantings during the summer rice growing seasons in Wuhan over two consecutive years (Table S2, Supplementary Table). The first planting, sown on May 15 or May 25 subjected the plants to natural long‐day conditions, whereas the second planting, sown on June 10 or June 15 exposed the plants to natural short‐day conditions. The results indicated that, regardless of whether the near‐isogenic lines ZS97 and NIL(NYZ) were sown in May or June, there were highly significant differences in grain protein content, with NIL(NYZ) consistently exhibiting lower values (Table S2, Supplementary Table). Furthermore, statistically significant differences in grain protein content were observed between ZS97^b^ and NIL(NYZ)^a^, as well as between ZS97^d^ and NIL(NYZ)^c^, both of which headed almost simultaneously (Table S2, Supplementary Table). These findings suggest that the regulation of grain quality by *Ghd7* may not be solely dependent on heading.

*Ghd7* is absent in ZS97 (*Ghd7*‐0).^[^ ^37^ ^]^ The *XI* accession Minghui 63 (MH63) carries a strong functional allele of *Ghd7* (*Ghd7*‐1).^[^ ^38^ ^]^ Transgenic positive plants carrying the *Ghd7*‐1 allele (CM+) exhibit significantly lower grain protein content compared to transgenic negative plants (CM‐). (Figure 1a; Figure S1d,f, Supporting Information). Zhonghua11 (ZH11), a *Geng*/*Japonica* (*GJ*) accession, has a weak functional allele of *Ghd7* (*Ghd7*‐2).^[^ ^39^ ^]^ *Ghd7* overexpressed plants had significantly lower grain protein content than wild type ZH11, whereas *Ghd7*‐silenced plants had significantly higher grain protein content than wild type ZH11. (Figure 1a; Figure S1d,e,g, Supporting Information).

**Figure 2:**
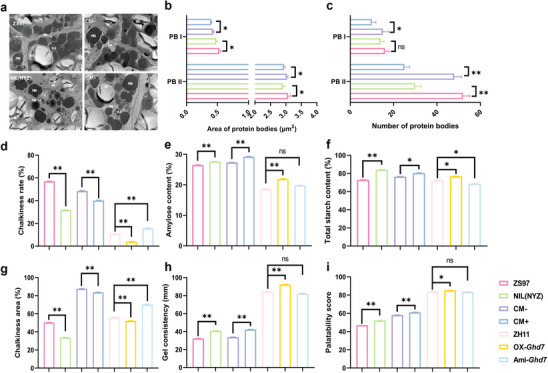
Effects of *Ghd7* on protein bodies and grain quality traits in NILs and transgenic plants. a) Ultrastructure of cells in the developing endosperm of NILs and transgenic plants at 15 DAF. PBI and PBII, protein bodies I and II; SG, starch granule; ER, endoplasmic reticulum; ECS, extracellular space; CW, cell wall. VLS, vesicle‐like structures. Scale bars, 2 µm. b) Mean area of protein bodies in the endosperm of NILs and transgenic plants. c) Number of protein bodies in the endosperm of NILs and transgenic plants. d, e) Chalkiness. f) Amylose content. g) Total starch content. h) Gel consistency. i) Palatability score. Data are means ± s.e.m (*n* = 3 in b and c; *n* = 16 in d, e, and f; *n* = 10 in g, h, and i). *, ** and ns indicate significant differences at *p *< 0.05, *p *< 0.01 and no significant difference respectively, *t*‐tests.

About 90% of grain protein is seed storage protein composed of albumins, globulins, prolamins, and glutelins.^[^ ^40^ ^]^ We assayed the seed storage protein contents in the NILs and transgenic plants. The glutelin content of NIL(NYZ) was 22.5 mg g^−1^ lower than that of ZS97, and the glutelin content of CM+ was 12.7 mg g^−1^ lower than that of CM‐ (Figure 1b). Although the contents of the other three proteins were very low, their levels in NIL(NYZ) and CM+ were significantly lower than in ZS97 and CM‐, respectively, except for the albumin between CM+ and CM‐ (Figure 1c–e). Similarly, all four seed storage proteins in OX‐*Ghd7* were significantly lower than in ZH11, whereas the contents of these proteins in Ami‐*Ghd7* were significantly higher than in ZH11 (Figure 1b–e). Most of the seed storage protein is in protein bodies (PB) in the endosperm. We employed transmission electron microscopy to determine the effect of *Ghd7* on PB. In the endosperm at 15 days after flowering (DAF), prolamin‐containing PB I and glutelin/globulin‐containing PB II were clearly discernible in ultrathin sections (**Figure** **2**a). Compared with the endosperm cells from ZS97 and CM‐, the endosperm cells from NIL(NYZ) and CM+ had greatly reduced numbers of PBI and PBII with decreased mean area (Figure 2a,b,c), indicating that *Ghd7* reduced the storage space for storage proteins, an intuitive cause of decreased protein content in the endosperm.

A genome‐wide association study of grain protein content^[^ ^41^ ^]^ detected three significant SNPs on chromosome 7 in *XI* subpopulation, and all three mapped close to the *Ghd7* locus (Figure S3 and Table S3, Supplementary Table). Eight allelic variants of *Ghd7* were identified in the association panel (Figure S4 and Table S4, Supplementary Table). Among the four most frequent alleles, accessions carrying *Ghd7*‐0 had the highest grain protein content, in agreement with its loss of function (Figure 1f). There was no difference in protein content between accessions having *Ghd7*‐1 with strong function and *Ghd7*‐4 with unknown function^[^ ^38^, ^42^ ^]^ (Figure 1f), implying that *Ghd7*‐4 was a strong functional haplotype of *Ghd7*. Varieties carrying *Ghd7*‐2 had a protein content higher than those of *Ghd7*‐1 and *Ghd7*‐4 but lower than that of *Ghd7*‐0 (Figure 1f). We compared the protein contents of the four most frequent alleles using seed storage protein data from the association panel.^[^ ^41^ ^]^ For glutelin content, all three functional *Ghd7* alleles (*Ghd7*‐1, *Ghd7*‐4, and *Ghd7*‐2) had lower values than *Ghd7*‐0, with *Ghd7*‐4 having the lowest value among the three (Figure 1g). *Ghd7*‐4 had the lowest prolamin and globulin contents, whereas *Ghd7*‐1 had a lower globulin content than *Ghd7*‐2 and *Ghd7*‐0 (Figure 1h,i). The albumin levels were not significantly different among the four alleles (Figure 1j).

All the data suggested that *Ghd7* was a negative regulator of grain protein content.

### 2.2 *Ghd7* Enhances Grain Quality

The reduction in grain protein content caused by *Ghd7* was expected to affect grain quality. We explored the effects of *Ghd7* on grain quality in the NILs and transgenic plants. Chalkiness rate and area of NIL(NYZ), CM+, and OX‐*Ghd7* were significantly lower than those of ZS97, CM‐, and ZH11, respectively, whereas the chalkiness of Ami‐*Ghd7* was significantly higher than that of ZH11 (Figure 2d,e). In contrast, amylose content, total starch content and gel consistency of NIL(NYZ), CM+ and OX‐*Ghd7* were higher than those of the respective controls, whereas no difference was observed in amylose content and gel consistency but significantly decrease in total starch between Ami‐*Ghd7* and ZH11 (Figure 2f,g,h). The effect of *Ghd7* on protein content, amylose content, starch content, and gel consistency prompted us to further investigate the palatability score of the NILs and transgenic plants. The palatability scores of NIL(NYZ), CM+, and OX‐*Ghd7* were significantly higher than those of their respective controls, and there was no difference between Ami‐*Ghd7* and ZH11 (Figure 2i). We also measured the fatty acid contents in the NILs and transgenic materials. Although there was no change in total fatty acid contents, the relative content of individual components did change (Table S5, Supplementary Table), possibly also contributing to changes in palatability score.

Considering that the *Ghd7* alleles carried by NIL(NYZ) and CM+ were *Ghd7*‐2 and *Ghd7*‐1, respectively, we also collected near isogenic lines carrying *Ghd7*‐4 alleles NIL(RH003) with 1892S background and investigated various quality traits (Table S6, Supplementary Table). Like the other two most widely distributed alleles, *Ghd7*‐4 also significantly improved the appearance quality and cooking and eating quality. We also analyzed phenotypic data^[^ ^43^, ^44^ ^]^ of 533 accessions from previous studies and found that *Ghd7*‐4 conferred no difference in yield compared with the known strong functional allele *Ghd7*‐1, but both were significantly higher than *Ghd7*‐2 and *Ghd7*‐0. Notably, rice varieties carrying the *Ghd7*‐4 allele exhibit a similar plant height to those carrying the *Ghd7*‐1 allele, yet they flower earlier than both *Ghd7*‐1 and *Ghd7*‐2 (Figure S5, Supporting Information), which is consistent with the findings reported by Lu et al.^[^ ^42^ ^]^

These results show that all three alleles of *Ghd7* (*Ghd7*‐1, *Ghd7*‐4, and *Ghd7*‐2) have pleiotropic effects on grain quality, simultaneously improving appearance and cooking and eating quality.

### 2.3 *Ghd7* Enhances NUE and Increases Grain Yield

As previous studies showed that *Ghd7* positively regulates nitrogen use efficiency^[^ ^45^, ^46^ ^]^ and yield,^[^ ^37^, ^45^ ^]^ we further investigated the performance of *Ghd7* at varying nitrogen fertilization levels (Figure S6a,b, Supporting Information). The values of four traits, including grain yield, dry matter weight, nitrogen content per plant, and grain nitrogen content per plant, increased with increasing nitrogen concentration in both NIL(NYZ) and ZS97, with all values in NIL(NYZ) significantly higher than those in ZS97 (Figure S6c–f, Supporting Information). Three measures of NUE, namely NUE for dry matter production, NUE for grain production, and agronomic nitrogen use efficiency, were calculated for the two NILs. Although NUE for dry matter production and grain production in the NILs decreased with increasing nitrogen concentration, both values for NIL(NYZ) were significantly higher than those for ZS97 (Figure 6g,h, Supporting Information). Compared with ZS97 plants, NIL(NYZ) plants exhibited significantly higher agronomic nitrogen use efficiency (Figure S6i, Supporting Information). Notably, the yield of NIL(NYZ) at 2N nitrogen concentration was 42.6% higher than that at 0N nitrogen, whereas the yield of ZS97 increased only by 32.77% with the same treatment (Figure S6c). These results indicated that *Ghd7* confers a substantial increase in yield with improved nitrogen use efficiency.

### 2.4 *Ghd7* Coordinates Carbon and Nitrogen Metabolism in Seedlings

**Figure 3:**
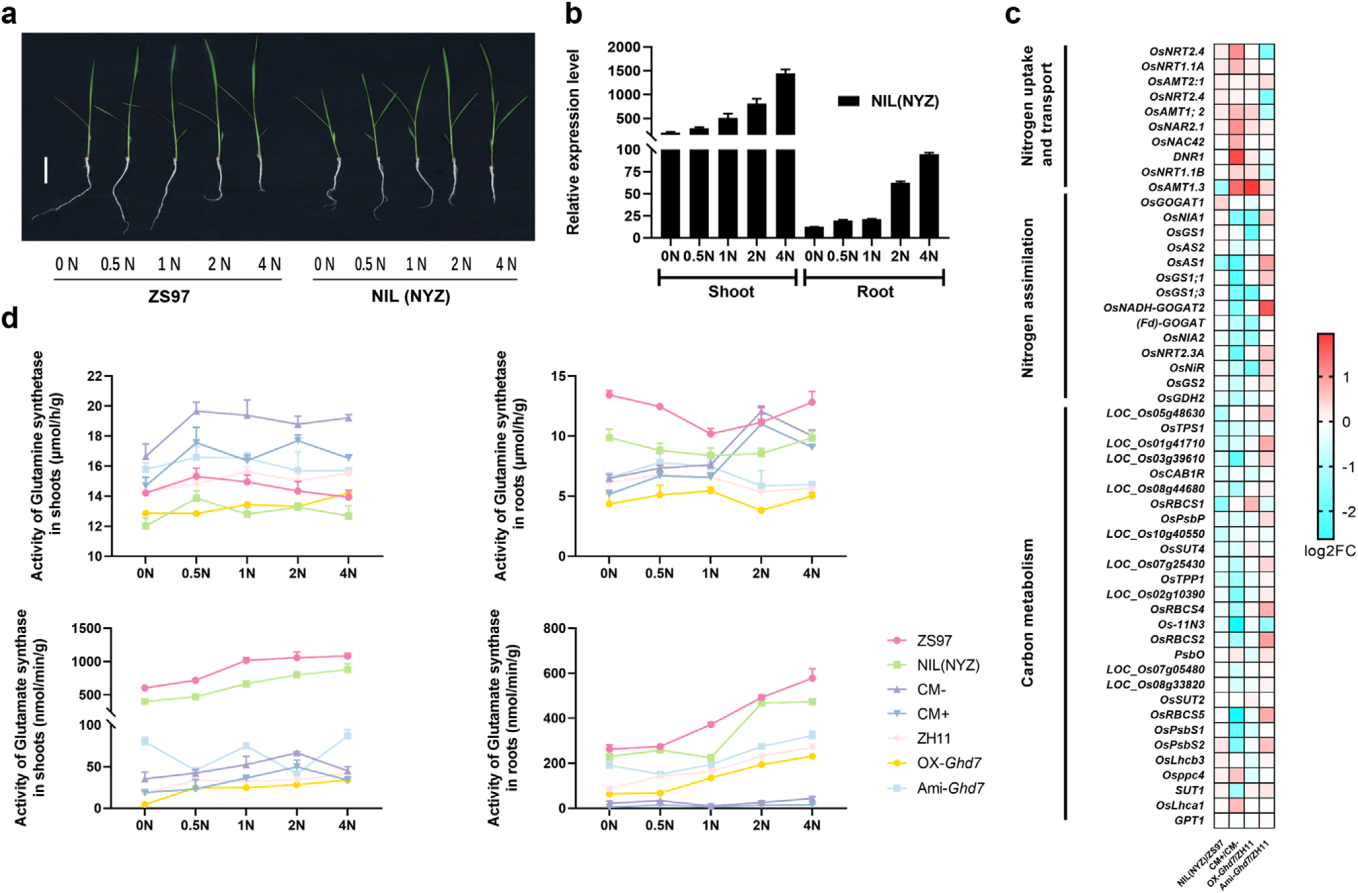
*Ghd7* inhibits nitrogen assimilation. a) Seedling length statistics of ZS97 and NIL(NYZ) at 15 days after germination under different nitrogen conditions. Bar, 5 cm. b) Expression levels of *Ghd7* in the shoot and root of NIL(NYZ) under different nitrogen concentrations. c) Relative expression analysis of carbon and nitrogen metabolism genes in 15‐day‐old seedlings of NILs and transgenic plants grown in the field under natural light conditions. d) Activities of glutamine synthetase (GS) and glutamate synthase (GOGAT) in the shoots and roots of NIL seedlings at 15 days after germination. *n* ≥ 9; Data are means ± s.e.m.

Reduced grain protein content caused by *Ghd7* may be an outcome of its regulation of nitrogen metabolism. We explored the regulation of nitrogen assimilation by *Ghd7* in seedlings based on its highest expression in leaves (Figure S7a, Supporting Information). A two‐week hydroponic experiment with a gradient of nitrogen concentrations was conducted using the two NILs (**Figure** **3**a). Both shoot length of NIL(NYZ) and expression level of *Ghd7* increased along with increasing nitrogen concentration (Figure 3b; Figure S7b, Supporting Information), indicating that *Ghd7* was positively responsive to nitrogen. We first analyzed expression levels of 28 genes^[^ ^47^, ^48^, ^49^, ^50^, ^51^, ^52^ ^]^ encoding nitrogen assimilation‐associated enzymes and transcription factors under control nitrogen condition (i.e., 1N nitrogen concentration); 13 genes exhibited significantly lower expression in the shoot of NIL(NYZ) than in ZS97, including *OsNiR*, *OsNAR2.1*, *OsGDH2*, *OsNADH‐GOGAT1*, *OsGS1;1*, *OsNADH‐GOGAT2*, *(Fd)‐GOGAT*, *OsGS1:2*, *OsNIA1*, *OsGS2*, *OsGDH1*, *OsAS1*, and *NIGT1*, and only *TOND1*, a low nitrogen tolerance gene,^[^ ^53^ ^]^ showed higher expression. In contrast, the expression levels of 5 genes (*OsNiR*, *OsNAR2.1*, *OsGS1:1*, *qSBM1*, and *TOND1*) were significantly higher in roots of NIL(NYZ) than in ZS97 (Figure S7c, Supporting Information). These results suggested that *Ghd7* may inhibit nitrogen assimilation in shoots at the seedling stage, but promote nitrogen absorption in the roots. Relative expression level comparison of ZS97 and NIL(NYZ) indicated that *Ghd7* upregulates most nitrogen transporters and downregulates most carbon metabolism genes, involving regulatory elements of photosynthesis (for example, *OsCAB1R* and *OsRBCS1*), sucrose metabolism (for example, *OsTPS1* and *OsTPP1*), and sucrose transport (for example, *OsSUT2* and *OsSUT4*), as well as most nitrogen assimilation genes (Figure 3c). We subsequently measured activities of various nitrogen assimilation‐related enzymes under all nitrogen conditions. The two key enzymes, glutamine synthetase (GS) and glutamate synthase (GOGAT), displayed significantly lower activities in NIL(NYZ), CM+ and OX‐*Ghd7* than in ZS97, CM‐, and ZH11 respectively, under all nitrogen concentrations in both shoots and roots, while the results of Ami‐*Ghd7* were the opposite. (Figure 3d). Similar results were also obtained for NADH‐glutamate dehydrogenase (NADH‐GDH), asparagine synthase (AS), and Nitrate reductase (NR) (Figure S7d, Supporting Information). These results indicated that nitrogen assimilation in rice seedlings was inhibited by *Ghd7*.

Seedling length of NIL(NYZ) was significantly shorter than that of ZS97 at the end of the two‐week culture period (Figure 3a; Figure S7b, Supporting Information), a pattern opposite to the final plant height of the NILs at maturity (Figure S1a, Supporting Information). Combined with previous research findings,^[^ ^39^, ^54^ ^]^ this is likely to be a result of Ghd7 dynamically regulating hormones in plants. Gene expression analysis of key genes involved in gibberellins (GAs) synthesis and abscisic acid (ABA) accumulation was conducted in 2‐week‐old and 1‐month‐old seedlings of NILs grown in the field (Figure S8, Supporting Information). The results showed that compared to ZS97 seedlings of the same age, the expression levels of genes *OsGA2ox1*, *OsGA2ox5*, *OsGA2ox10*, and *RACK1A* were significantly lower in 2‐week‐old NIL(NYZ) seedlings (Figure S8a, Supporting Information). However, in one‐month‐old seedlings, except for *sd37*, the expression levels of other genes (including *GID2*, *OsGA2ox5*, *PHYC*, *SLR1*, and *RACK1A*) were higher in NIL (NYZ) (Figure S8b, Supporting Information). Additionally, we measured the endogenous active GA_3_ levels in the shoots of 2‐week‐old and one‐month‐old NIL seedlings. Our results indicated that the endogenous active GA_3_ level in NIL (NYZ) at two weeks was significantly lower than that of ZS97. Conversely, at one month of age, the endogenous active GA_3_ level in NIL (NYZ) was higher than that of ZS97 (Figure S8c, Supporting Information). Therefore, this particular time point can be considered as a critical turning point where Ghd7 regulates plant hormone levels and begins to positively influence plant growth and development. Certainly, this time point may vary depending on the genetic background. We then conducted a one‐month hydroponic experiment with a gradient of nitrogen concentrations (Figure S8d, Supporting Information). At the end of one‐month culture, NIL(NYZ) approximated ZS97 in shoot and root lengths (Figure S8d,e, Supporting Information), and NIL(NYZ) significantly exceeded ZS97 in total nitrogen contents of the tissues under all nitrogen concentrations except 0N (Figure S8f, Supporting Information). We assayed abundances of mRNAs of genes encoding NH_4_^+^‐uptake transporters and NO_3_^−^‐uptake transporters under different nitrogen conditions in roots of 2‐week‐old using reverse transcription quantitative PCR (RT–qPCR) (Figure S8g, Supporting Information). Notably, most of the genes detected showed higher expression in NIL(NYZ) than in ZS97 under all nitrogen conditions. In comparison to ZS97 and CM‐ plants without *Ghd7*, NIL(NYZ) and CM+ plants with *Ghd7* exhibited a stronger capacity for nitrogen absorption. Similarly, when compared to the wild type ZH11, the nitrogen absorption rate in OX‐*Ghd7* plants was significantly increased, whereas that in Ami‐*Ghd7* plants was decreased (Figure S8h, Supporting Information). Taken together, these nitrogen transport genes may have already played an active role in the earlier stage of rice growth and development under the regulation of *Ghd7*, which led to the counterattack of NIL(NYZ) in the later stage.

Carbon and nitrogen metabolisms are often intricately related and mutually regulated during plant growth and development. To examine the effect of *Ghd7* on carbon metabolism, we firstly determined the expression of key genes in the carbon metabolism pathway,^[^ ^55^, ^56^ ^]^ including the Rubisco (Ribulose bisphosphate carboxylase oxygenase) family, the phosphoenolpyruvate carboxylase (PEPC) family and sugar transportation‐related genes, using the NILs from one‐month hydroponic culture under different nitrogen conditions (Figure S9a, Supporting Information). *Ghd7* seemed to be more inclined to promote the expression of sugar transport‐related genes than photosynthetic carbon metabolism genes, especially under 0.5N and 2N conditions (Figure S9a, Supporting Information). We then measured the activities of key enzymes, Rubisco and PEPC (Figure S9b,c, Supporting Information). The activity of Rubisco in NIL(NYZ) was significantly lower than that in ZS97 under low nitrogen concentrations (0N and 0.5N), but significantly higher under control (1N) and high (2N and 4N) nitrogen concentrations. The Rubisco activity of CM+ was higher than that of CM‐ at 0.5N, 1N, and 4N nitrogen concentrations, while the enzyme activity of OX‐*Ghd7* was higher than that of ZH11 at 0N and 4N nitrogen concentration (Figure S9b, Supporting Information). PEPC activities in NIL(NYZ), CM+, and OX‐*Ghd7* were significantly lower than in ZS97, CM‐, and ZH11, respectively, under 0.5N, but were significantly higher at 2N (Figure S9c, Supporting Information), with no difference observed at other concentrations. The C:N ratio, an index used to monitor carbon and nitrogen metabolism in plants,^[^ ^29^ ^]^ in NIL(NYZ) was significantly lower than in ZS97 at all nitrogen concentrations except for 4N, at which the ratio in NIL(NYZ) was significantly higher (Figure S9d, Supporting Information). The comparison of C:N ratios between OX‐Ghd7 and ZH11 showed similar results to those of the near‐isogenic lines, whereas CM+ exhibited higher values at 0N, 1N, and 4N nitrogen concentrations (FigureS9d, Supporting Information). These results suggest that *Ghd7* plays different roles in coordination of carbon and nitrogen metabolism at different nitrogen concentrations.

**Figure 4:**
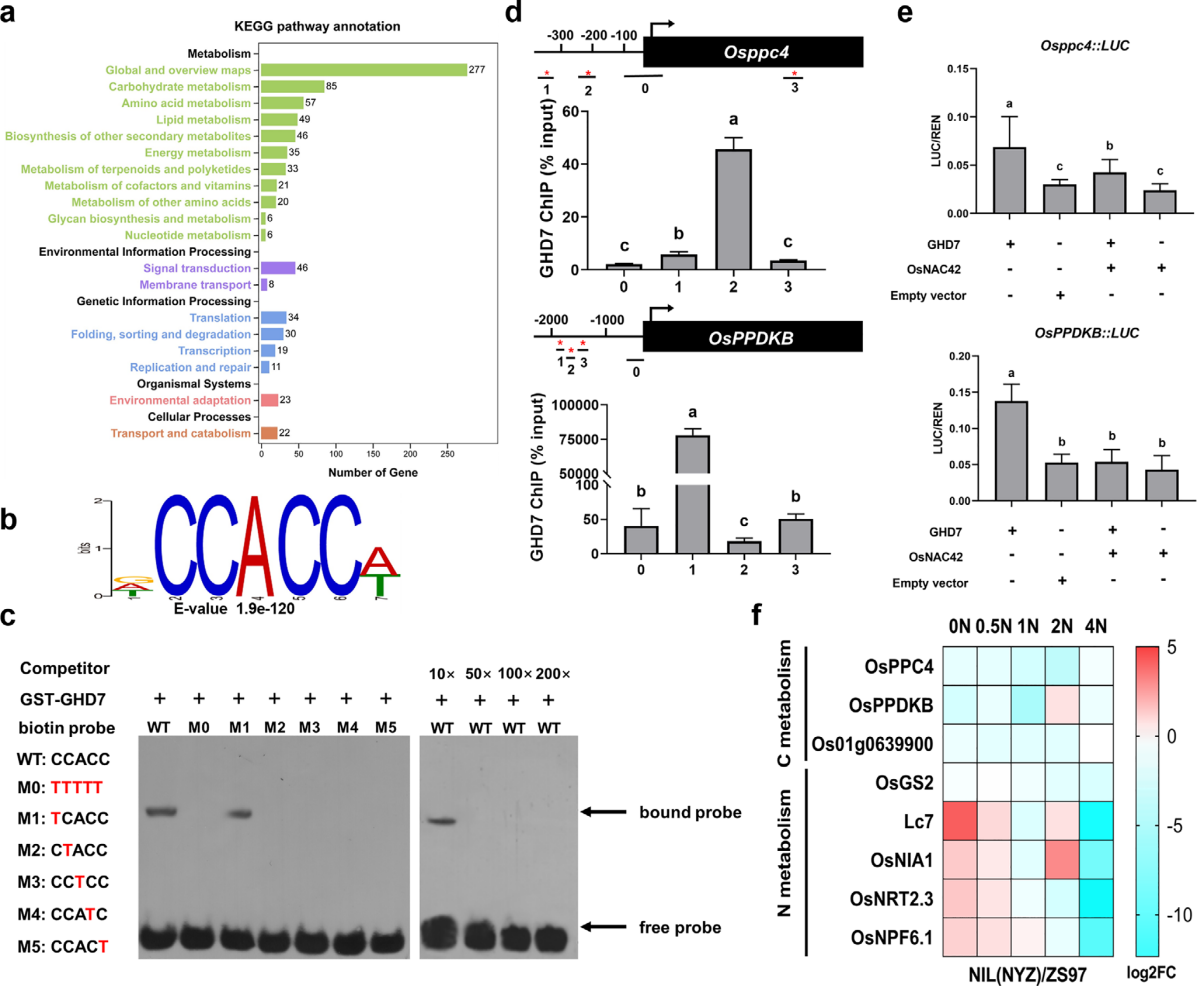
*Ghd7* coordinates carbon and nitrogen metabolism in the seedling. a) Kyoto Encyclopedia of Genes and Genomes (KEGG) analysis of the downstream potential target genes regulated by Ghd7 in the seedling shoot. b) Sequence motif enriched in ChIP–seq with Ghd7 in seedlings. c) EMSA assays. Unlabeled probes were used as competitors. Red marks are the mutated bases in the core motif. d) Ghd7‐mediated ChIP–qPCR enrichment (relative to input) of CCACC‐containing fragments (marked with an asterisk) from *Osppc4*, *OsPPDKB*. e) Transactivation assays in rice protoplasts. f) Expression levels of target genes regulated by Ghd7 directly under different nitrogen concentrations, data are means ± s.e.m (*n* = 3). In (d) and (e), different letters denote significant differences (*p* < 0.05) from a Duncan's multiple range test.

DNA sequencing of Ghd7 chromatin‐immunoprecipitation products (ChIP–seq) and Kyoto Encyclopedia of Genes and Genomes (KEGG) pathway analysis revealed that downstream potential target genes regulated by Ghd7 were significantly enriched in three items, namely “Global and overview maps”, “Amino acid metabolism” and “Carbohydrate metabolism” (**Figure** **4**a). Moreover, a predominant CCACC motif is shared by multiple carbon and nitrogen metabolism genes (Figure 4b). Electrophoretic mobility shift assays (EMSA) demonstrated that GST‐Ghd7 recombinant protein was capable of efficient binding to biotin‐labeled DNA fragments containing intact but not mutant CCACC core motifs, and the binding activity was inhibited by biotin‐free probes in a dose‐dependent manner (Figure 4c). ChIP–qPCR confirmed in vivo association of Ghd7 with CCACC‐containing nucleotide fragments from multiple carbon and nitrogen metabolism genes, including *Osppc4*, *OsPPDKB*, *Os01g0639900*, *OsGS2*, *Lc7*, *OsNIA1*, *OsNRT2.3*, and *OsNPF6.1* (Figure 4d; Figure S10, Supporting Information). Further experiments showed that Ghd7 trans‐activated transcription of *Osppc4*, *OsPPDKB*, *Os01g0639900*, *OsGS2*, *Lc7*, *OsNIA1*, *OsNRT2.3*, and *OsNPF6.1* (Figure 4e; Figure S11, Supporting Information). In particular we found that the expression of four nitrogen metabolism genes (*Lc7*, *OsNIA1*, *OsNRT2.3*, and *OsNPF6.1*) was significantly upregulated by Ghd7 under low nitrogen conditions (0N and 0.5N) (Figure 4f), consistent with the results of protoplast transcriptional activity assays (Figure 4e; Figure S11, Supporting Information), but not under other nitrogen conditions. This indicated that the regulation of Ghd7 on nitrogen metabolism genes is affected by nitrogen levels.

### 2.5 *Ghd7* Coordinates Carbon and Nitrogen Metabolism in Endosperm

**Figure 5:**
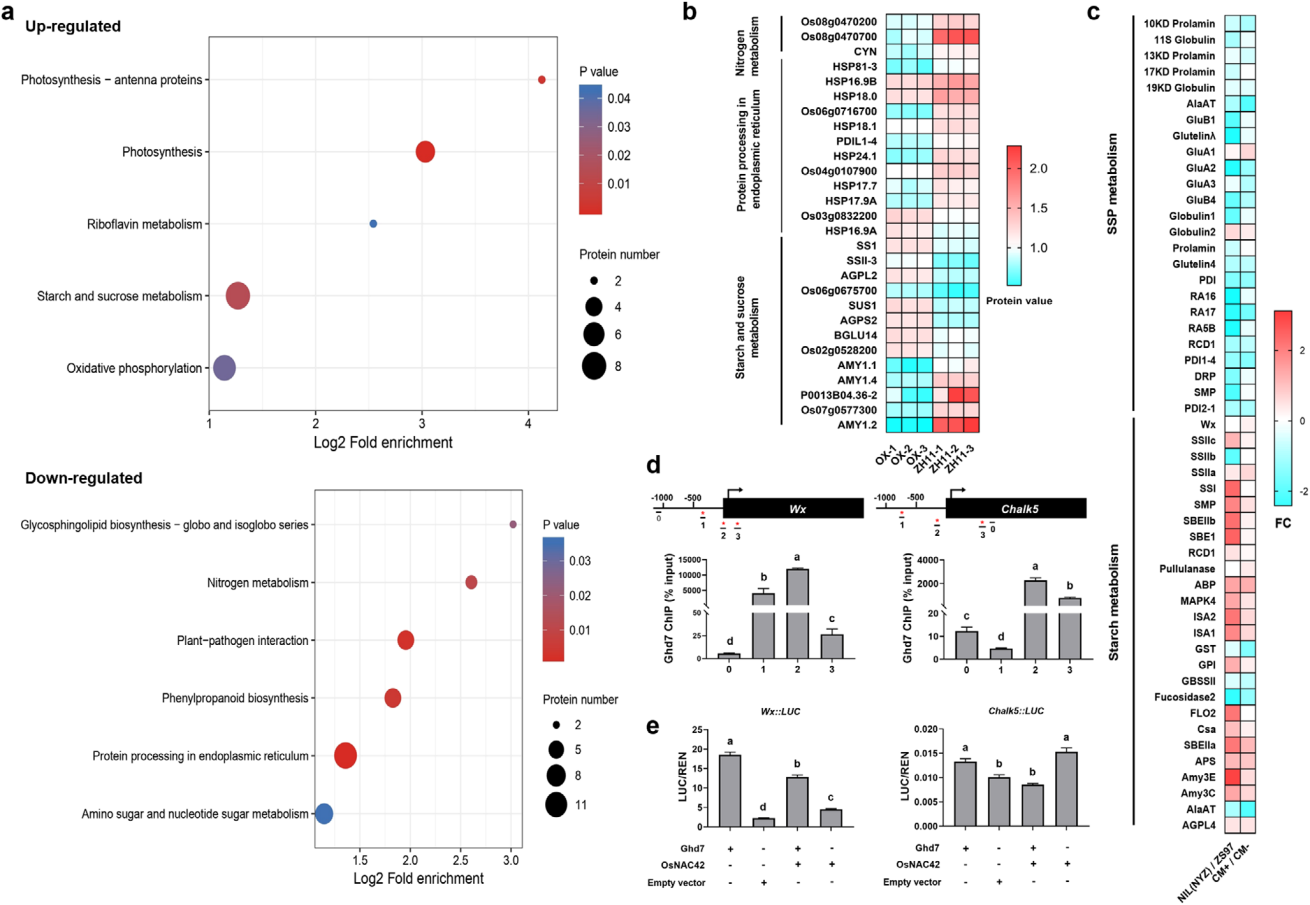
*Ghd7* coordinates carbon and nitrogen metabolism in endosperm. a) Bubble diagram of enrichment and distribution of differentially expressed proteins in the KEGG pathway. b) Heatmap of abundance of 28 quantifiable DEPs identified in the OX‐*Ghd7* and ZH11 proteomic data. c) A heatmap of key genes involved in the synthesis and storage of endosperm components in NILs and transgenic complementary lines. The colour key (sky blue to red) represents gene expression as fold change of (NIL(NYZ)/ZS97) or (CM+/CM‐). d) Ghd7‐mediated ChIP–qPCR enrichment (relative to input) of CCACC‐containing fragments (marked with an asterisk) from *Wx* and *Chalk5*. DNA fragments not marked with an asterisk and numbered 0 do not contain the CCACC motif. For genes containing more than three CCACC motifs, three DNA fragments containing the CCACC motif were randomly selected for enrichment analysis. e) Transactivation assays in rice protoplasts. In *d* and *e*, different letters denote significant differences (*p* < 0.05) from a Duncan's multiple range test.

In order to explore the effect of *Ghd7* in coordinating carbon and nitrogen metabolism in the endosperm, iTRAQ (isobaric tags for relative and absolute quantification) quantitative proteomics was carried out for seed samples 25 DAF from OX‐*Ghd7* and ZH11 to detect differentially expressed proteins (DEPs). DEPs down‐regulated by *Ghd7* were mainly distributed in six pathways, while DEPs up‐regulated by *Ghd7* were significantly enriched in another five pathways (**Figure** **5**a). Notably, among quantifiable DEPs identified, most nitrogen metabolism‐related proteins (involved in nitrogen metabolism and protein processing in the endoplasmic reticulum) were significantly down‐regulated in OX‐*Ghd7* relative to ZH11, whereas most carbon metabolism‐related proteins (involved in starch and sucrose metabolism) were significantly up‐regulated (Figure 5a,b). Correspondingly, qPCR analysis showed that in genetic materials carrying *Ghd7*, the transcripts of most genes related to seed storage protein metabolism were downregulated, whereas transcripts of most genes related to starch metabolism were upregulated (all detected genes were referenced to previous reports^[^ ^18^, ^57^ ^]^) (Figure 5c). Like the results from seedlings, ChIP‐seq analysis revealed that the target genes regulated by Ghd7 in endosperm were also mainly enriched in “Global and overview maps”, “Amino acid metabolism”, and “Carbohydrate metabolism” (Figure S12, Supporting Information). ChIP‐qPCR assays confirmed that Ghd7 protein was capable of efficiently enriching multiple carbon and nitrogen metabolism genes that contain CCACC motifs in vivo, including *Wx*, *Chalk5*, *OsGS1;3*, and *OsNIA1* (Figure 5d; Figure S10, Supporting Information). Further experiments demonstrated that Ghd7 promoted the transcriptional activities of these target genes (Figure 5e; Figure S11, Supporting Information). However, transcription of some target genes, such as *OsNIA1*, related to nitrogen assimilation, was repressed (Figure S7c, Supporting Information), suggesting that Ghd7 eventually exhibited inhibitory effects with the help of other interacting factors.

### 2.6 OsNAC42 is a Key Balancer that Helps Ghd7 Maintain Superior Quality and High Yields

**Figure 6:**
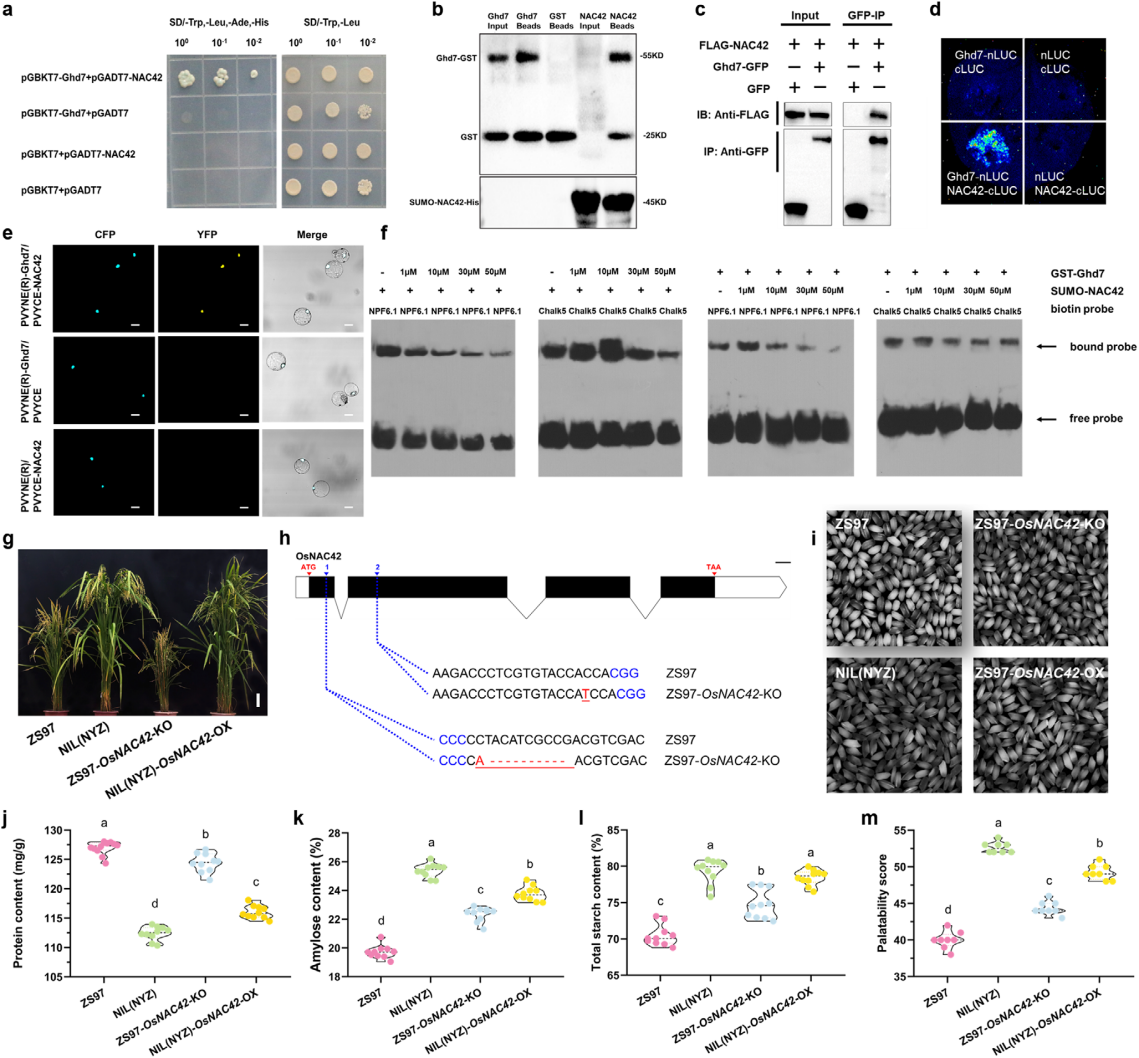
Ghd7 interacts with OsNAC42. a) Ghd7 interacts with OsNAC42 in yeast cells. Yeast cells were cultured on SD/‐Trp‐Leu or SD/‐Trp‐Leu‐His‐Ade media, and X‐α‐Gal was added to SD/‐Trp‐Leu‐His‐Ade medium. 10^−0^, 10^−1^ and 10^−2^ represent no dilution, 10‐fold dilution and 100‐fold dilution, respectively. AD, GAL4 activation domain; BD, GAL4 DNA‐binding domain; SD, synthetic dropout. b) In vitro pull‐down assay proved the direct physical interaction between Ghd7 and OsNAC42. c) Coimmunoprecipitation assay of Ghd7 and OsNAC42 interaction in rice cells. Ghd7‐GFP was cotransfected with FLAG‐OsNAC42. d) Split firefly luciferase complementation (SFLC) assays. nLUC‐tagged Ghd7 was co‐transformed into tobacco leaves along with the cLUC‐targeted OsNAC42. nLUC and cLUC represent two empty vectors. e) Confirmation of the Ghd7 and OsNAC42 interaction by BiFC assay in rice protoplasts. Representative cells are shown, as imaged by laser‐scanning confocal microscopy. Detection of PVYNE(R)‐Ghd7 and PVYCE‐NAC42 interaction in rice protoplasts is shown as a yellow signal. The cyan signal was the nuclear location protein Ghd7:CFP. The empty vector was co‐expressed with each recombinant vector and used as a control. Bars, 10 µm. f) EMSA assays. The DNA fragments come from the promoters of *OsNPF6.1* and *Chalk5* and were labeled with biotin at the 5′ end. g) Plant architecture of NILs, the mutant of *OsNAC42* gene knockout in ZS97 genetic background (ZS97‐*OsNAC42*‐KO) and *OsNAC42* gene overexpressed mutant in NIL(NYZ) genetic background (NIL(NYZ)‐*OsNAC42*‐OX). Scale bar, 10 cm. h) Schematic diagram of the targeted sites in *OsNAC42*. Blue numbers 1 and 2 represent editing targets 1 and 2, respectively. Mutated sites are underlined, and the PAMs are highlighted in blue. The deleted sequences are shown by red hyphens. Bar, 100 bp. i) Grain chalkiness of NILs, ZS97‐*OsNAC42*‐KO and NIL(NYZ)‐*OsNAC42*‐OX mutant plants. Main grain quality traits in NILs, ZS97‐*OsNAC42*‐KO and NIL(NYZ)‐*OsNAC42*‐OX mutant plants, including grain protein content j), amylose content in grain k), total starch content in grain l), and palatability score m). In (j‐m), data are means ± s.e.m (*n* = 20). Different letters denote significant differences (*p* < 0.05) from a Duncan's multiple range test.

In order to identify interacting proteins that assist Ghd7 in coordinating carbon and nitrogen metabolism, we carried out a yeast two‐hybrid screen and found OsNAC42, a transcription factor belonging to the NAC family (**Figure** **6**a). Interaction between Ghd7 and OsNAC42 was confirmed by in vitro pull‐down assays, in vivo co‐immunoprecipitation (Co‐IP), split firefly luciferase complementation (SFLC) in tobacco leaf epidermal cells, and biomolecular fluorescent complementation (BiFC) in rice protoplasts (Figure 6b–e). Ghd7 alone can activate the transcription of downstream carbon and nitrogen metabolism genes in seedlings and endosperm, while OsNAC42 plays a completely opposite role, and the intensity of transcription activity decreases to some extent when they coexist. (Figures 4e and 5e; Figure S11, Supporting Information). *OsNPF6.1*, a downstream target gene of Ghd7 (Figure 4c; Figures S10 and S11, Supporting Information), was also directly regulated by OsNAC42^[^ ^58^ ^]^ (Figure S13, Supporting Information). Moreover, *Chalk5*,^[^ ^57^ ^]^ a negative regulator of total grain protein content, was not only regulated by Ghd7 (Figure 4c; Figure 5d,e), but also confirmed to be a downstream target gene of OsNAC42 (Figure S13, Supporting Information). Therefore, *OsNPF6.1* and *Chalk5* were used as representative target genes of the Ghd7‐OsNAC42 complex for further analysis. The EMSA assays demonstrated that the binding ability of OsNAC42 protein to *OsNPF6.1* would gradually weaken by maintaining the OsNAC42 protein level constant while gradually increasing the amount of Ghd7 protein (Figure 6f). Similarly, by keeping the protein level of Ghd7 constant and gradually increasing the protein amount of OsNAC42, the binding ability between Ghd7 protein and *OsNPF6.1* would also gradually weaken (Figure 6f). Regarding *Chalk5*, when the level of OsNAC42 protein was kept constant and the amount of Ghd7 protein was gradually increased, the binding between OsNAC42 and *Chalk5* initially strengthened and then weakened (Figure 6f). Conversely, a similar trend was observed when the protein level of Ghd7 was fixed and the amount of OsNAC42 protein was gradually increased (Figure 6f). This suggests that the Ghd7‐OsNAC42 complex may have different regulatory modes for different target genes, and the binding ability with downstream target genes is affected by the antagonism of this complex.

In order to further clarify the role of OsNAC42 in the regulation of downstream target genes by Ghd7, we acquired an *osnac42* mutant with a loss‐of‐function SNP mutation (Ala405 to Val) in ZH11 (Figure S14a,b, Supporting Information). Expression of *OsNAC42* was dramatically decreased in the *osnac42* mutant relative to the wild type (Figure S14c, Supporting Information). Transcriptome analysis revealed significant up‐regulation of *OsGS1;3* expression in the endosperm of Ami‐*Ghd7* (Figure S14d, Supporting Information). Meanwhile, the expression of *Chalk5* and *OsNIA1* was down‐regulated, while the expression level of *Wx* remained relatively stable. In the endosperm of OX‐*Ghd7*, there was a significant up‐regulation of *Wx*, *OsNIA1*, and *OsGS1;3* expression, while *Chalk5* expression was significantly down‐regulated (Figure S14d, Supporting Information). In contrast to *Ghd7*, the mutation of the *OsNAC42* gene did not cause drastic fluctuations in the expression of the four downstream target genes, although *Wx*, *OsNIA1*, and *OsGS1;3* still showed up‐regulation (Figure S14d, Supporting Information). Similar to the situation in the endosperm, although the expression of some target genes in *osnac42* mutant seedlings changed and had statistical differences, they did not fluctuate too sharply (Figure S14e, Supporting Information). Half of the genes, including *Osppc4*, *OsPPDKB*, *Os01g0639900*, and *OsNPF6.1*, were down‐regulated, while only *OsNIA1* was up‐regulated, and the expression levels of *OsGS2*, *Lc7*, and *OsNRT2.3* remained almost unchanged (Figure S14e, Supporting Information). In addition, the expression levels of some target genes in OX‐*Ghd7* and Ami‐*Ghd7* also changed to varying degrees, but not as violently as in the endosperm. Taken together, all these results suggest that the regulation of Ghd7‐OsNAC42 complex varies with different downstream target genes.

After analyzing the key agronomic characteristics, it was found that compared with ZH11, the plant height, number of panicles, number of effective panicles, and yield per plant of the *osnac42* mutant were significantly reduced, while thousand‐grain weight were significantly increased, and the heading date was extended by about two days (Table S7, Supplementary Table). Further rice quality analysis revealed lower protein in *osnac42* mutant grains, while total starch content, amylose content in the grain, and palatability score were higher than those of ZH11 (Figure S15a, Supporting Information). Furthermore, we also overexpressed *OsNAC42* driven by the maize ubiquitin promoter in Nipponbare to investigate the effects of elevated expression of *OsNAC42* on rice yield and quality (Figure S14f,g, Supporting Information). *OsNAC42* expression level in OX‐*OsNAC42* was elevated approximately 30‐fold compared with WT (Figure S14h, Supporting Information). Although the number of panicles in OX‐*OsNAC42* plants increased, the effective number of panicles did not increase accordingly. Furthermore, OX‐*OsNAC42* plants exhibited significantly lower plant height, number of grains per panicle, panicle length, and yield per plant compared to ZH11. Similar to *osnac42* mutants, the heading date of OX‐*OsNAC42* plants was also delayed by approximately two days (Table S7, Supplementary Table). Evaluation of rice quality showed that compared with ZH11, the grain protein content of OX‐*OsNAC42* increased, but the amylose content in grain, total starch content in grain, and palatability value decreased (Figure S15b, Supporting Information). These results indicated that OsNAC42 is likely to be a balancer in the process of Ghd7 maintaining high yield and superior quality in rice.

To further elucidate the genetic relationship between Ghd7 and OsNAC42, we generated the *OsNAC42* gene knockout mutants with ZS97 genetic background (ZS97‐*OsNAC42*‐KO) and *OsNAC42* gene overexpressed mutants with NIL(NYZ) genetic background (NIL(NYZ)‐*OsNAC42*‐OX) (Figure 6g,h; Figure S14f, Supporting Information). Agronomic trait analysis revealed that ZS97‐*OsNAC42*‐KO plants exhibited the lowest values for plant height, number of grains per panicle, panicle length, and yield per plant (Figure 6g; Table S8, Supplementary Table). In contrast, NIL(NYZ)‐*OsNAC42*‐OX plants demonstrated the highest number of panicles per plant and effective panicle number per plant, and had no significant difference from NIL(NYZ) in plant height, heading date, number of grains per panicle, panicle length and 1000‐grain weight (Figure 6g; Table S8, Supplementary Table). However, despite the observed increase in the number of panicles per plant and the effective panicle number per plant, the yield per plant of NIL(NYZ)‐*OsNAC42*‐OX was reduced due to a significant decrease in the seed setting rate (Table S8, Supplementary Table). Additionally, we measured the quality traits of ZS97, NIL(NYZ), ZS97‐*OsNAC42*‐KO, and NIL(NYZ)‐*OsNAC42*‐OX using data from a field trial in 2024. Compared with ZS97, ZS97‐*OsNAC42*‐KO had a 34.98% lower chalkiness rate, a 20.23% lower chalkiness area, a 2.04% decrease in protein content (mainly glutelin), a 13.33% increase in amylose content, a 6.11% increase in total starch content and a 10.94% increase in palatability value (Figure 6i‐m; Figure S15c, Supporting Information). We also compared these traits between NIL(NYZ) and NIL(NYZ)‐*OsNAC42*‐OX, but observed completely opposite results (Figure 6i‐m; Figure S15c, Supporting Information). Taken together, OsNAC42 not only helps maintain stable yield with Ghd7, but also antagonizes Ghd7 and jointly determines the taste quality of rice.

## 3 Discussion

As a negative regulator of rice protein content, the variation in *Ghd7* coding region endows rice with different grain protein content and eating quality. Notably, different alleles of *Ghd7* can improve rice quality under different genetic backgrounds. Among them, *Ghd7*‐4 has the lowest glutelin, prolamin, and globulin content and shortened growth period, and the yield performance is not inferior to the currently known strong functional haplotype *Ghd7*‐1. Haplotype analysis showed that *Ghd7*‐4 was mainly distributed in indica rice varieties, accounting for about 1/4 of all rice varieties, and did not exist in japonica rice varieties (Figure S4, Supporting Information). Therefore, introducing the superior allele into japonica rice varieties may be a highly promising strategy to improve the quality of japonica rice.

Ghd7 strongly inhibits nitrogen assimilation in rice seedlings and prevents excessive nitrogen distribution into grains, which may be the root cause of the decrease in grain protein content. After all, Ghd7's inhibition of nitrogen assimilation is absolute, while other metabolic processes, including nitrogen absorption and transport, and carbon metabolism, can be flexibly adjusted according to the received signal. Therefore, we believe that this is more likely to be a strategy or mechanism of Ghd7 to maintain the optimal growth and development state of rice at all times.

Weng et al reported that Ghd7 may function as a sensor for the plant to adapt to dynamic environmental inputs.^[^ ^39^, ^59^ ^]^ Coincidentally, in this study, we found that Ghd7 also functions as a sensor of nitrogen signaling. Under low‐nitrogen conditions, Ghd7 inhibits nitrogen assimilation and carbon metabolism but promotes nitrogen uptake and transport; under high‐nitrogen conditions, Ghd7 inhibits nitrogen assimilation and nitrogen uptake and transport, but promotes carbon metabolism. Furthermore, the absence of the functional module Ghd7‐OsNAC42 significantly impairs the rice plant's responsiveness to nitrogen as well as its absorption capacity (Figure S16, Supporting Information). Previous studies have shown that Ghd7 itself can function as either a transcriptional repressor or a transcriptional activator.^[^ ^39^, ^60^ ^]^ In our study, although Ghd7 appears as a transcriptional activator, its ability to respond to changes in the external nitrogen supply level and maintain the balance of carbon and nitrogen metabolism in rice cannot be achieved by its simple transcriptional activation or transcriptional repression. Therefore, we speculate that Ghd7 may respond to environmental factors, including nitrogen signals, and dynamically regulate carbon and nitrogen metabolism with the assistance of other interaction factors.

OsNAC42 is such a Ghd7‐interacting factor, which forms a heterodimeric complex with Ghd7 and antagonizes each other to regulate downstream target genes to achieve optimal carbon and nitrogen homeostasis in rice. Judging from the results of transcriptional activity analysis alone, OsNAC42 seems to be a pure inhibitor, and the transcriptional activation ability of Ghd7 to downstream target genes is always weakened when it exists. When *OsNAC42* gene was mutated, in addition to *Chalk5*, the expression levels of *Wx*, *OsNIA1*, and *OsGS1;3* were all up‐regulated in the endosperm, which also supports this view. In *osnac42* seedlings, half of the eight downstream target genes were down‐regulated, only *OsNIA1* was significantly up‐regulated, which may mean that OsNAC42 acts as a transcriptional activator for carbon and nitrogen metabolism target genes in seedlings, which is consistent with the conclusion of Tang et al.^[^ ^58^ ^]^ OsNAC42 and Ghd7 are both transcription factors with both transcriptional activation and transcriptional repression functions. The interaction between them gives rice more flexibility in regulating its C/N balance, such as different regulation modes for target genes involved in different metabolic processes.

**Figure 7:**
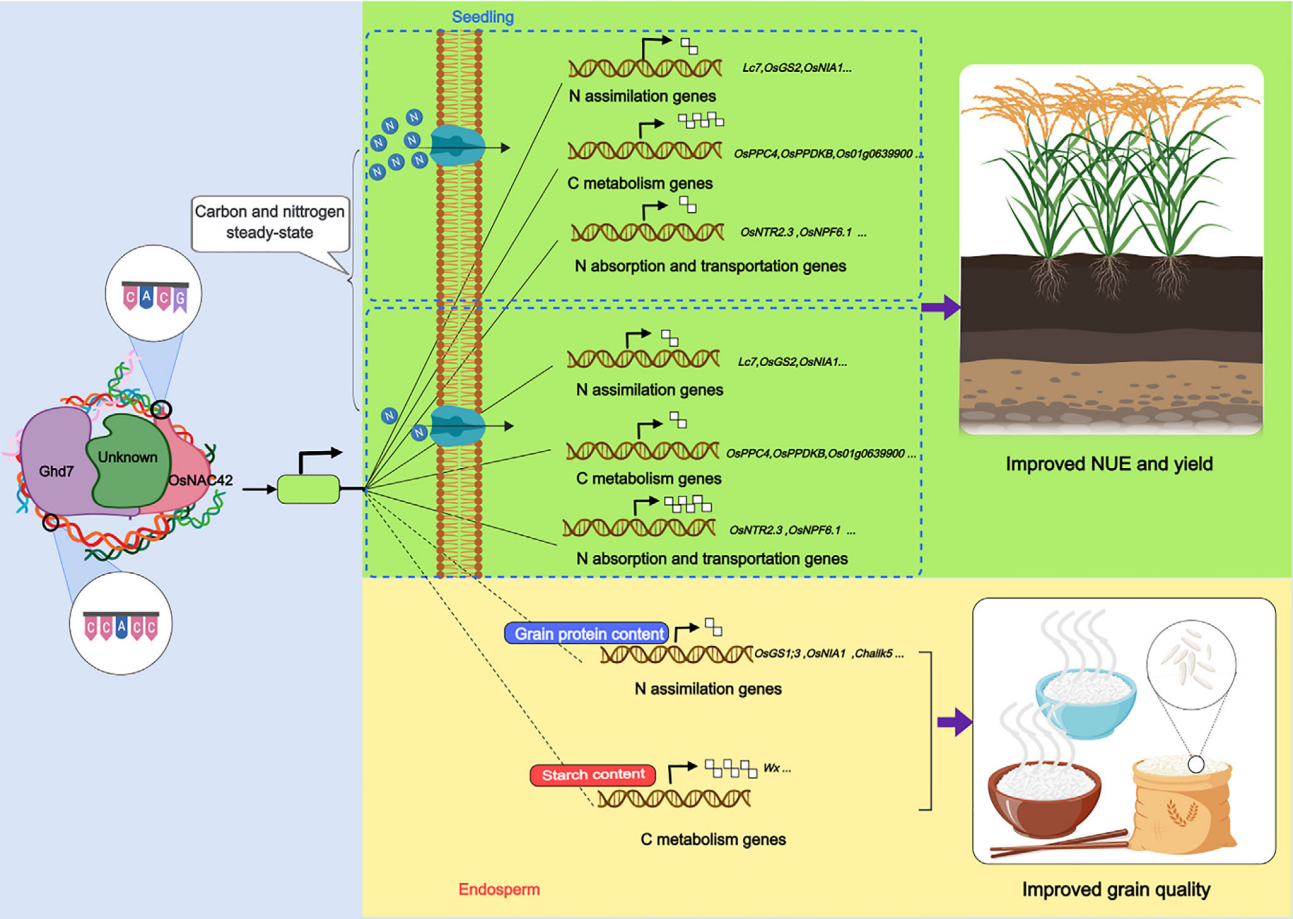
Proposed model of Ghd7‐centered complex regulating carbon and nitrogen metabolism in seedlings and endosperm to synergistically improve rice yield and quality. The complex centered on Ghd7 binds to numerous carbon and nitrogen metabolism genes on the genome through the corresponding motifs and transactivates the transcriptional expression of these carbon and nitrogen metabolism genes. With the assistance of OsNAC42 and other unknown Ghd7‐interacting proteins, this complex can regulate different metabolic processes in different tissues in different ways and respond to external environmental signals. In seedlings, when cells sense low‐nitrogen signals, this complex inhibits nitrogen assimilation and carbon metabolism, but enhances nitrogen uptake and transport; when cells sense high‐nitrogen signals, this complex inhibits nitrogen assimilation and nitrogen uptake and transport, but enhances carbon metabolism. In the endosperm, this complex inhibits the expression of nitrogen assimilation‐related genes, such as OsGS1;3, but promotes the expression of carbon metabolism‐related genes, such as Wx, thus changing the composition of the grain (increased starch and decreased protein). This flexible regulatory mechanism of Ghd7 keeps the carbon and nitrogen metabolism in rice always in a dynamic balance and maintains optimal growth and development status, which is also the key to maintaining high quality and high yield of rice.

In the endosperm, OsNAC42 inhibits the transcriptional activation activity of Ghd7 on “carbon” and “nitrogen” metabolism genes to a certain extent, thereby affecting the grain protein and amylose contents that determine rice quality at an appropriate level. In other words, Ghd7 negatively regulates grain protein content and positively regulates grain amylose content, while OsNAC42 has the opposite effect. Therefore, this antagonistic relationship between Ghd7 and OsNAC42 helps to maintain the storage substances in grains at an appropriate level, so as to achieve fine regulation of rice eating quality. In this process, Ghd7 may be a central regulator, while the role of OsNAC42 is more like a balancer, which can help Ghd7 maintain C/N balance, thereby achieving synergy between superior quality and high yield. In addition, there must be other Ghd7 interaction factors (including direct and indirect interactions) involved in the dynamic regulation of nitrogen signal response and C/N balance, and the possible working modes are shown in **Figure** **7**. Weng et al.^[^ ^39^ ^]^ and Wang et al.^[^ ^59^ ^]^ reported that Ghd7 is a central regulator of growth, development, and stress response, which coincides with our results. Ghd7 is known for its pleiotropy, affecting many traits including heading date, yield, number of grains per panicle, grain size, nitrogen use efficiency, chlorophyll content, vascular bundle variation, zinc content in brown rice, and stress response.^[^ ^24^, ^37^, ^39^, ^60^, ^61^, ^62^, ^63^, ^64^ ^]^ Now we reveal the new function of Ghd7 in negative regulation of grain protein content and the positive regulation of rice quality. Taken together, all these may be the reflection of Ghd7 as a central regulatory factor in the regulatory network of carbon and nitrogen metabolism. In a word, our work reveals that Ghd7 is a green super rice (GSR) gene that enables rice to achieve both high yield and superior quality, and deciphers the mechanism by which Ghd7 can maintain both high yield and superior quality in rice, providing new insights and ideas for future coordination of high yield and quality rice production.

## 4 Experimental Section

### Genetic Materials

The coding regions from ZS97 and NYZ cDNAs to construct overexpression vectors for *LOC_Os07g15820* were each amplified by primer OX and ligated into the vector pCAMBIAl301U under the control of the maize ubiquitin promoter. The two overexpression vectors were transformed into ZH11, and the resulting lines carrying the coding region from ZS97 were named as OX‐1^ZH11^ and that from NYZ was named as OX‐2^ZH11^. To generate the OX‐*OsNAC42* overexpression vector, the full‐length *OsNAC42* coding region was isolated from ZH11 genomic DNA and inserted into the binary vector pU2301‐flag, which was transformed into Nipponbare.

Transgenic materials for *Ghd7*, including ZS97, NIL(NYZ), CM+, CM‐, OX‐*Ghd7* and Ami‐*Ghd7*, were described previously.^[^ ^36^, ^39^ ^]^

The tilling mutant (*osnac42*) was obtained from the Crop Tilling Mutant Database of Prof. Chunming Liu, Key Laboratory of Plant Molecular Physiology, CAS (http://www.croptilling.org).^[^ ^65^ ^]^

Primers used in vector construction and mutant screening are listed in Table S9 (Supplementary Table).

### Allele Analysis of *Ghd7*

Allele analysis of *Ghd7* was performed on a panel of 533 rice accessions. Twelve SNPs in the coding sequence of *Ghd7* identified in the panel were downloaded from RiceVarMap 2.0 (http://ricevarmap.ncpgr.cn/v2/) and subjected to allele classification; eight alleles were identified after referring to the articles by Lu et al^[^ ^42^ ^]^ and Zhang et al^[^ ^38^ ^]^ (Figure S4 (Supporting Information) and Table S10, Supplementary Table).

### Field Planting and Quality Trait Measurement

Field experiments were conducted in experimental fields of Huazhong Agricultural University at Wuhan (Hubei Province) and Lingshui (Hainan Province). Germinated seeds were sown in mid‐May each year and transplanted after 25 days. Plants were spaced 16.5 cm apart in rows separated by 26 cm. Field management followed local agricultural practices. Harvested grains were air‐dried and stored at room temperature for at least 3 months before evaluating quality traits. Well‐filled grains were used to measure all quality characteristics. Brown rice was used to evaluate total protein^[^ ^66^ ^]^ and fatty acid contents; milled rice was used to assess chalkiness rate and area, and palatability score; and milled rice flour was used to determine amylose,^[^ ^67^ ^]^ storage protein contents,^[^ ^3^, ^41^ ^]^ and gel consistency.^[^ ^67^ ^]^

### Field Nitrogen Treatment Assay

Three nitrogen treatments, low (0N, 0 kg ha^−1^), control (1N, 124.5 kg ha^−1^) and high (2N, 249 kg ha^−1^) nitrogen, were applied to NIL (ZS97) and NIL (NYZ). There were three replications for each NIL and each nitrogen treatment, arranged in a randomized block design. Basic P and K fertilizers for each treatment were P_2_O_5_ at 75 kg ha^−1^ and K_2_O at 75 kg ha^−1,^ respectively. Urea (46% N), superphosphate (P_2_O_5_ 12%) and potassium chloride (K_2_O 60%) were used as fertilizer sources. Among them, 5/13 of the nitrogen fertilizer was base fertilizer, 4/13 was applied at tillering, and 4/13 at flowering; 50% of the phosphorus of potassium fertilizers were applied as base fertilizers.

Nitrogen absorption and utilization were calculated as: nitrogen utilization efficiency for dry matter production: (NUEd, g/g) = Total plant dry matter /total plant nitrogen; and nitrogen utilization efficiency for grain production (NUEg, g/g) = Grain yield /total plant nitrogen content.

### Hydroponic Culture and Enzyme Activity Assays

To determine the effects of *Ghd7* on nitrogen assimilation in seedlings, a standard rice culture solution for hydroponic experiments was used [1.44 mM NH_4_NO_3_, 0.3 mM NaH_2_PO_4_, 0.5 mM K_2_SO_4_,1.0 mM CaCl_2_, 1.6 mM MgSO_4_, 0.17 mM Na_2_SiO_3_, 50 µM Fe‐EDTA, 0.06 µm (NH_4_)_6_Mo_7_O_24_, 15 µM H_3_BO_3_, 8 µM MnCl_2_, 0.12 µM CuSO_4_, 0.12 µM ZnSO_4_, 29 µM FeCl_3_, and 40.5 µM citric acid with pH adjusted to 5.5 by sulfuric acid].^[^ ^46^ ^]^ After germination, rice seedlings were grown in nitrogen‐deficient (0N), 0.5‐fold nitrogen (0.5N), normal nitrogen (1N), 2‐fold nitrogen (2N) and 4‐fold nitrogen (4N) nutrient solutions, which were renewed every 5 days. One batch of NILs and transgenic materials was grown in a greenhouse for two weeks, and a different batch was grown under natural light conditions for one month. Rice seedlings were grown in the greenhouse under long days (14:10 h, light: darkness at 30 °C) photoperiods at 70% humidity or grown outside under natural conditions (June / July). In the subsequent seedling nitrogen treatment experiment, the nutrient solution compositions under different nitrogen supply levels were as follows: 0N, no nitrogen added; LN, 0.15 mM NH_4_NO_3_; HN, 2.5 mM NH_4_NO_3_. Shoot and root lengths of seedlings were determined using ImageJ software (US National Institutes of Health). Except for samples used to determine nitrate reductase activity (Must be fresh samples), plant tissues (Including the shoot tissue and the root tissue of the seedlings) from two‐week‐old seedlings were immediately frozen in liquid nitrogen after sampling and stored at −80 °C before use. Activities of various enzymes (including GS, NADH‐GOGAT, NADH‐GDH, AS, NiR, NR, Rubisco, PEPC) related to carbon metabolism and nitrogen assimilation were determined using kits purchased from Grace Biotechnology Company (Suzhou, China). Taking the NADH‐GOGAT enzyme activity measurement as an example, the principle and measurement process were as follows: GOGAT catalyzes the transfer of the amino group of glutamine to α‐ketoglutarate, forming two molecules of glutamic acid; at the same time, NADH is oxidized to generate NAD+, and the absorbance at 340 nm decreases. The rate can reflect the activity of GOGAT. Preheat the microplate reader for more than 30 min, adjust the wavelength to 340 nm, and zero with distilled water. Add 9 mL of reagent 1 to reagent 2, dissolve and mix thoroughly, place in a 25 °C water bath for 5 min; add 20 µL of sample and 180 µL of reagent 2 to the 96‐well plate, mix, and immediately record the absorbance value A1 at 340 nm for 20 s and the absorbance value A2 after 5 min 20 s, calculate ΔA = A1‐A2. Finally, the activity calculation formula of NADH‐GOGAT enzyme is NADH‐GOGAT(nmol/min/g FW) = [ΔA×V_0_/(ε×d) ×10^9^] / (W×V_1_/V_2_)/T = 643×ΔA /W. V_0_, total volume of reaction system, 2 × 10^−4^ L; V_1_, added sample volume, 0.02 mL; V_2_, the volume of extraction liquid added, 1 mL. ε, NADH molar extinction coefficient, 6.22 × 10^3^ L mol cm^−1^; d, 96‐well plate optical path, 0.5 cm; T, reaction time, 5 min.

And all other enzyme activities were determined according to the manufacturer's instructions. Absorbance values were detected using a TECAN Infinite M200 microplate reader. For both expressional and enzyme activity analysis, three plants (or tissues from three plants) were mixed as a replication, and three biological replicates were conducted for each line.

### ^15N^ Accumulation Assay

Rice seedlings were cultured in International Rice Research Institute nutrient solution (1.44 mM NH_4_NO_3_) for 3 weeks, with water change once a day. Uniformly growing seedlings were selected for further treatment. Then, the seedlings were pretreated with 2 mM (NH4)_2_SO4 and 2 mM KNO_3_ for 1 week, and transferred to a nitrogen‐free solution for starvation treatment for 4 days. They were then transferred to 0.1 mM CaSO4 for 1 minute and treated with 1.46 mM ^15^N_2_‐labeled NH_4_NO_3_ (98 atom% % ^15^N; Sigma; cat. no. 366528‐1G) for 10 minutes. Finally, rinse the roots of the seedlings with 0.1 mM CaSO4 solution and deionized water, and collect them. The samples were dried at 70 °C for 7 days and then ground to fine powder for ^15^N‐content detection by an isotope ratio mass spectrometer with an elemental analyser (Thermo Finnigan Delta Plus XP; Flash EA 1112).

### Nitrogen Concentration Assays and Analysis of C/N Ratio

Shoot and root samples from rice seedlings were collected and placed in a dryer at 80 °C for 2 days, and then ground into powder for determining total nitrogen content by a Kjeldahl nitrogen analyzer. Soluble sugar contents in related seedling tissues were determined using kits purchased from Grace Biotechnology Company (Suzhou), and the ratio of soluble sugar content to nitrogen content was used to represent the C:N ratio.

### RNA Reverse Transcription and qRT‐PCR

Total RNA from plant tissues was isolated using Trizol reagent (Invitrogen); 4 µg of RNA was treated with DNaseI and then reverse‐transcribed with M‐MLV reverse transcriptase (Promega Madison, WI, USA) and Oligo (dT) 18 primers (Promega). The concentration of cDNA was measured using a NanoDrop2000 (Thermo Scientific) and diluted to 10 ng µL, which was used for subsequent qPCR amplification. The qPCR amplification mixture contained 6 µl template cDNA, 5 µL 2× FastStart Universal SYBR Green Master (ROX) Mix (ROCHE), and 0.25 mM forward and reverse primers. Amplifications were carried out on an ABI7500 PCR instrument (Applied Biosystems). The rice constitutive expression gene *Actin1*, was used as the internal control. Primers used are listed in Tables S11–S15 (Supplementary Table).

### RNA‐seq Analysis

Shoot samples of rice seedlings grown in the field for one month under natural light conditions were collected, quickly frozen in liquid nitrogen, and then transferred to an ultra‐low temperature refrigerator for storage until use. Seeds 14 days after flowering were collected, carefully peeled off the chaff with tweezers, quickly placed the endosperm in liquid nitrogen, and then transferred them to an ultra‐low temperature refrigerator until use. RNA extraction, library construction, and sequencing were performed by the Novogene Institute (Novogene, Tianjin, China). The clustering of the index‐coded samples was performed on a cBot Cluster Generation System using TruSeq PE Cluster Kit v3‐cBot‐HS (Illumia)

According to the manufacturer's instructions. After cluster generation, the library preparations were sequenced on an Illumina Novaseq platform, and 150 bp paired‐end reads were generated. Raw data (raw reads) of fastq format were first processed through FastP software. In this step, clean data (clean reads) were obtained by removing reads containing adapter, reads containing poly‐N, and low‐quality reads from raw data. At the same time, Q20, Q30, and GC content of the clean data were calculated. All the downstream analyses were based on the clean data of high quality. Reference genome and gene model annotation files were downloaded from the genome website directly (http://rice.uga.edu/pub/data/Eukaryotic_Projects/o_sativa/annotation_dbs/pseudomolecules/version_7.0/all.dir/all.con; http://rice.uga.edu/pub/data/Eukaryotic_Projects/o_sativa/annotation_dbs/pseudomolecules/version_7.0/all.dir/all.gff3). The Index of the reference genome was built using Hisat2 v2.0.5, and paired‐end clean reads were aligned to the reference genome using Hisat2 v2.0.5. Hisat2 was selected as the mapping tool for that Hisat2 can generate a database of splice junctions based on the gene model annotation file and thus a better mapping result than other non‐splice mapping tools. featureCounts v1.5.0‐p3 was used to count the read numbers mapped to each gene. And then FPKM of each gene was calculated based on the length of the gene and the read count mapped to this gene. FPKM, expected number of Fragments Per Kilobase of transcript sequence per Millions base pairs sequenced, considers the effect of sequencing depth and gene length for the reads count at the same time, and is currently the most commonly used method for estimating gene expression levels. Differential expression analysis of two groups (two biological replicates per condition) was performed using the DESeq2 R package (1.20.0). DESeq2 provides statistical routines for determining differential expression in digital gene expression data using a model based on the negative binomial distribution. The resulting *p*‐values were adjusted using Benjamini and Hochberg's approach for controlling the false discovery rate. Genes with an adjusted *p*‐value ≤0.05 found by DESeq2 were assigned as differentially expressed.

### Transmission Electron Microscopy Analyses

Tissue samples were cut into small pieces (<2 mm^3^), immediately put into 2.5% glutaraldehyde solution and fixed for at least 12 h, before observation by transmission electron microscopy (H‐7650, Hitachi). The number and area of protein bodies in each sample were measured by ImageJ software (US National Institutes of Health).

### Macromolecule Interaction Assay

Yeast‐two‐hybrid (Y2H) assays were performed using the ProQuest Two‐Hybrid System (Invitrogen). The *Ghd7* (CCT domain) coding region was cloned into the pGBKT7 (BD) vector, and the full‐length CDS of *OsNAC42* amplified from Nipponbare cDNA was cloned into the pGADT7 (AD) vector. The AD and BD constructs were co‐transformed into yeast strain AH109 and grown on synthetic dropout medium minus Leu and Trp (SD‐LT). To confirm the interaction, positive yeast clones were selected from the SD‐LT medium and then grown on synthetic dropout medium minus Leu, Trp, His, and Ade (SD‐LTHA) with X‐α‐Gal for at least three days.

Full‐length CDS of *Ghd7* for pull‐down assays was fused to the prokaryotic expression vector pGEX‐4T‐1 to express Ghd7‐GST. The full‐length CDS of *OsNAC42* was inserted into pATX‐SUMO to express SUMO‐NAC42‐His. Plasmids were transformed into *E. coli* strain BL21. Fusion proteins were induced with 0.5 mM IPTG at 37 °C for 4 h. Pull‐down assays were performed as reported previously.^[^ ^68^ ^]^ Anti‐His (Medical Biological Laboratories, 1:2000) and anti‐GST (GenScript, 1:5000) antibodies were used in immunoblotting analysis.

For bimolecular fluorescence complementation (BiFC) assays, the full‐length *OsNAC42* coding sequence and the full‐length *Ghd7* coding sequence were each cloned into the pVYCE and pVYNE(R) vectors according to a previous report.^[^ ^69^ ^]^ Rice shoot protoplasts were prepared as reported previously^[^ ^70^ ^]^ and 5 µg of each of the paired fusion vectors was mixed and co‐transformed into rice protoplasts prepared from leaf sheaths of ZS97 seedlings, together with Ghd7‐CFP as a marker for nuclear localization. The fluorescence signal in the transformed protoplast was imaged by confocal laser scanning microscopy (Olympus, FV1200).

Full‐length cDNAs of *OsNAC42* and *Ghd7* for SFLC assays were amplified, and the amplicons were inserted into pCAMBIA1300‐35S‐Cluc‐RBS and pCAMBIA1300‐35S‐HA‐Nluc‐RBS vectors, respectively. Detailed experimental procedures followed a previous report.^[^ ^71^ ^]^

### Electrophoretic Mobility Shift Assay (EMSA)

A LightShift Chemiluminescent EMSA Kit (Pierce Biotechnology) was used in electrophoretic mobility shift assays (EMSA). Oligonucleotide probes were synthesized and labeled with biotin by ThermoFisher Scientific. Ghd7‐GST and SUMO‐NAC42‐His fusion proteins were expressed in *E. coli* strain BL21. The recombinant protein was purified using either IDA‐Nickel Beads (BEAVER) or amylose resin (BioLabs) affinity chromatography. EMSA was performed according to the manufacturer's instructions (Thermo, No. 20 148).

### Transactivation Activity Assays

For transactivation activity assays, the promoters of target genes from Nipponbare, ZS97, or ZH11 were cloned into the pGreenII 0800‐LUC vector^[^ ^72^ ^]^ to analyze transcriptional activity, and the Renilla luciferase gene was used as an internal transformation control to provide an estimate of the extent of transient expression in the same construct. Full‐length cDNA of *OsNAC42* was amplified and inserted into the None vector^[^ ^73^ ^]^ to generate effector plasmid vectors. Transactivation analyses were performed using rice protoplasts as described for BIFC. The rice protoplasts were transfected with different combinations of vectors overnight, then harvested and lysed for detection of firefly luciferase activity (Promega, E1960), according to the manufacturer's recommendations. Relative luciferase activity was defined by the ratio of LUC to Ren activity.

The primers used in the above macromolecule interaction experiments are listed in Table S16 (Supplementary Table).

### Proteomic Analysis

Seed samples at 25 days after flowering were quickly frozen in liquid nitrogen and stored at −80 °C before use. The methods for peptide preparation and protein abundance profiling by mass spectrometry were as described previously.^[^ ^74^, ^75^, ^76^, ^77^ ^]^ TEAB‐dissolved peptide segments (0.5 m) were labeled according to the operation manual for the TMT kit and classified by high pH reverse HPLC on an Agilent 300Extend C18 instrument (5 µm particle size, 4.6 mm inner diameter, 250 mm length). The EASY‐nLC 1000 UPLC system was used to separate the peptides dissolved in the mobile phase A for liquid chromatography, and then the peptides were injected into the NSI ion source for ionization, followed by Q Exactive HF‐X mass spectrometry analysis. The MS/MS data were processed using MaxQuant Search Engine (v.1.5.2.8).

### ChIP Assays and KEGG Analysis

ChIP assays were performed as previously described with minor modifications.^[^ ^78^ ^]^ Approximately 5 g of two‐week‐old MH63 seedling samples and endosperm samples at 14 days after pollination were collected and fixed with 1% (v/v) formaldehyde in a vacuum at 20–25 °C for 15 min, and then homogenized in liquid nitrogen. After separating and lysing the nucleus, the chromatin complexes were separated and ultrasonically broken into fragments with an average size of 500 bp. Immunoprecipitations were carried out overnight at 4 °C using commercial anti‐Ghd7 antibodies (ABclonal, A20327). The precipitated DNA was recovered, dissolved, and stored at ‐70 °C. Illumina sequencing libraries were constructed according to the manufacturer's instructions, and then sequenced on a BGISEQ‐500 platform. Mapping sequencing reads to MH63 reference genome^[^ ^79^, ^80^ ^]^ using SOAP aligner/soap2.^[^ ^81^ ^]^ Using peak summits to define the peak location types on the genome, and motif search and classification were carried out as described before.^[^ ^82^ ^]^ Purified DNA samples were also used as templates for qPCR. The primers are listed in Table S15 (Supplementary Table). Genome (KEGG) analysis of the DEGs was implemented to identify significantly enriched metabolic pathways using the KEGG (Kyoto, Japan) Orthology program (http://kobas.cbi.pku.edu.cn/).^[^ ^83^ ^]^ We used the p‐value calculated by hypergeometric tests and corrected by FDR, taking FDR ≤0.05 as a threshold to identify significant functional categories and metabolic pathways.

### Statistical Analysis

Experimental data were analyzed using Excel 2016 or IBM SPSS Statistics 22.0 for basic statistical analysis, including t tests, correlation coefficients, and ANOVA. One‐way ANOVA (one‐way analysis of variance) and Duncan's multiple range tests were used to assess significant differences in data.

### Supplementary Note—Map‐Based Cloning of *qPC7*

In order to fine map *qPC7* we developed markers C26 and S72 to replace flanking markers MRG186 and MRG4499, and then identified 56 recombinants from three segregating populations totaling 20000 plants (Figure S2a,b, Supporting Information). We performed progeny tests on all 56 recombinants, developed eight new markers for genotyping recombinants, and finally delimited *qPC7* to a region of 96.6 kb between markers C26 and S72, still with the six recombinants (Figure S2c (Supporting Information) and Table S17, Supplementary Table). Sixteen ORFs were annotated within the bracketed region according to the reference Nipponbare genome (Table S18, Supplementary Table). Among them, *LOC_Os07g15770*, also known as *Ghd7*, was previously reported as an important regulator of heading date and yield potential,^[^ ^37^ ^]^ *LOC_Os07g15820* encodes an expressed protein, and the remaining ORFs encode a hypothetical protein, five transposon proteins, and eight retrotransposon proteins. Therefore, *Ghd7* and *LOC_Os07g15820* were considered candidates for *qPC7*. The primers used in map‐based cloning are listed in Table S9 (Supplementary Table).

*Ghd7* is absent in ZS97.^[^ ^37^ ^]^ Sequence analysis of *LOC_Os07g15820* revealed that it contained two exons and one intron, and there were 16 SNPs between the alleles from NYZ and ZS97 (Figure S17, Supporting Information). The CDS of *LOC_Os07g15820* from ZS97 was 369 bp, whereas that from NYZ had a deletion of one base at position 304 (in the second exon), resulting in a frameshift mutation. We then assayed the temporal and spatial expression patterns of *Ghd7* and *LOC_Os07g15820*. Among the nine tested tissues from NIL(NYZ), *Ghd7* displayed the highest expression levels in leaves, low levels in stems, leaf sheaths, endosperm, and root, and almost no expression in other tissues (Figure S7a, Supporting Information). *LOC_Os07g15820* displayed a constitutive expression pattern (Figure S18a, Supporting Information), and no difference was observed in the endosperms of the NILs at 7 and 14 days after flowering (Figure S18b, Supporting Information), indicating that the expression level of *LOC_Os07g15820* might not be correlated with grain protein content.

In order to verify the functional gene underlying *qPC7*, we conducted a series of transgenic tests. Transgenic results for *Ghd7* are provided in the main text. Transgenic positive ZH11 plants carrying a p35S::c*LOC_Os07g15820* ^NYZ^ construct (with the *LOC_Os07g15820* CDS from NYZ under control of the 35S promoter) displayed no difference in grain protein content relative to negative plants, and the same was true for transgenic positive ZH11 plants carrying a p35S::c *LOC_Os07g15820* ^ZS97^ construct and negative plants (Figure S18c, Supporting Information). Therefore, *Ghd7* was regarded to be the functional gene underlying *qPC7*.

### Genome‐Wide Association Study of Grain Protein Content

In order to identify novel genes conferring grain protein content, we performed a genome‐wide association study of grain protein content using a germplasm panel of 533 rice accessions originating from a worldwide geographic range.^[^ ^41^ ^]^ Protein contents of milled rice from the accessions grown in 2015 followed a normal distribution and displayed variation ranging from 88.84 to 121.96 mg g^−1^ (Figure S19, Supporting Information). A GWAS of grain protein content identified significant SNP_S_ in the whole population, *XI*, and *GJ* subpopulations (Figure S3, Supporting Information). A total of 32 loci affecting protein content and having phenotypic variation greater than 10% were distributed on all 12 chromosomes except 5, 8, and 10 (Table S3, Supplementary Table).

## Conflict of Interest

The authors declare no conflict of interest.

## Author Contributions

G. L. and P. C. contributed equally to this work (co‐first authors). G. L. and P. C. performed most of the experiments; H.G., J.X., S.W., Y.Z., Y.W., M.A., Y.C., J.B., X.T., and W.R. participated in part of phenotyping, genotyping, and biochemical experiments; L.W. provided assistance in the collection of genetic materials. G.L., P.C., and P.L. performed various data analyses. G.G. and Q.L.Z. participated in field experiments. B.W., H.Z., and Y.L. provided guidance for the determination of some quality traits. Y.H. designed experiments. G.L. and Y.H. wrote the manuscript; Q.F.Z., P.L. and X. L.  improved the manuscript; all authors discussed and commented on the manuscript. G.L. and P.C. contributed equally to this work.

## Supporting information



Supporting Information

Supplementary Table

## Data Availability

The data that support the findings of this study are available in the supplementary material of this article.
