# Supplementary material for: Antagonistic Ghd7‐OsNAC42 Complexes Modulate Carbon and Nitrogen Metabolism to Achieves Superior Quality and High Yield in Rice
Source: Adv Sci (Weinh). 2025 Jun 10;12(31):e04163. doi: 10.1002/advs.202504163 (PMC12376526; doi:10.1002/advs.202504163)
Supplement: Supplementary file 1 — Supporting Information [file ADVS-12-e04163-s002.docx]

Supplementary Materials for

Antagonistic Ghd7-OsNAC42 complexes modulate carbon and nitrogen metabolism to achieves high yield and superior quality in rice

Guangming Lou*, Pingli Chen*, Pingbo Li, Haozhou Gao, Jiawang Xiong, Shanshan Wan, Yuanyuan Zheng, Yufu Wang, Mufid Alam, Yingnanjun Chen, Lei Wang, Jingjing Bai, Xuan Tan, Wenting Rao, Bian Wu, Hao Zhou, Yanhua Li, Guanjun Gao, Qinglu Zhang, Jinghua Xiao, Xianghua Li, Xuelei Lai, Qifa Zhang, Yuqing He†

* These authors contributed equally to this work.

†Corresponding author. Email: yqhe@mail.hzau.edu.cn (YH).

**This WORD file includes:**

Supplementary Figure 1 to 19

Supplementary Note

**Other Supplementary Materials for this manuscript include the following:**

Supplementary Data 1 to 18 (Excel format)

Supplementary Figures


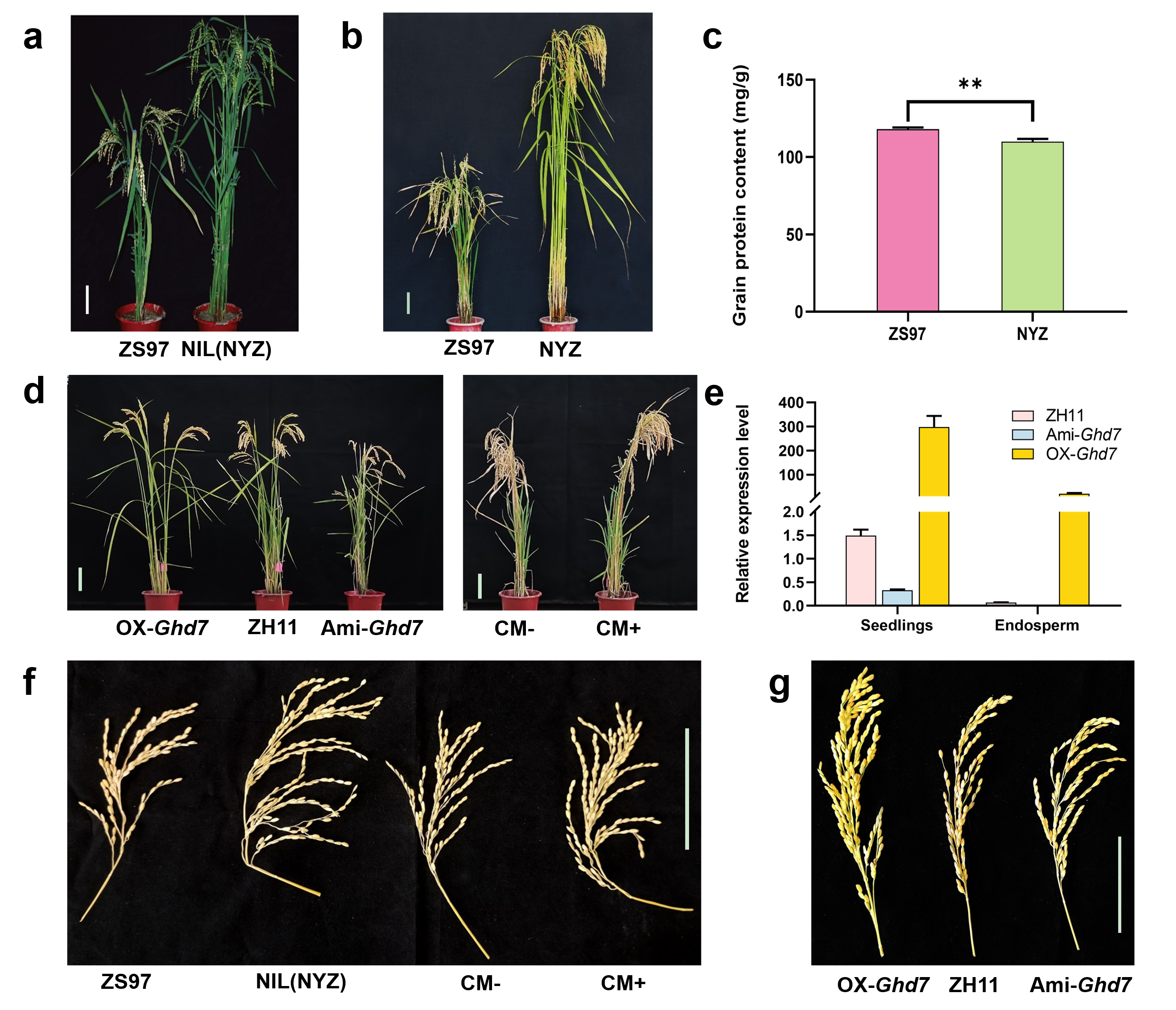


**Supplementary Figure 1 Phenotypes of *Ghd7*-related genetic material.** Plant architecture of NILs (**a**) and donor parents (**b**). Bar, 10 cm. NIL, near-isogenic line. (**c**) Protein contents of the parents. Data are means ± s.e.m (*n* = 20). **, significantly different at *P* <0.01, *t*-test. (**d**) Plant architecture of *Ghd7* transgenic materials. Bar, 10 cm. (**e**) Relative expression levels of *Ghd7* in the seedlings and endosperm of transgenic materials. Total RNA was extracted from the shoots of one-month-old seedlings and the endosperm after 15 days filling. qRT-PCR technology was used to detect expression levels. *Actin*1 was used as an internal reference gene. (**f**) Panicles of NILs and complementary materials. Bar, 10 cm. (**g**) Panicles of *Ghd7* transgenic materials and ZH11. Bar, 10 cm.


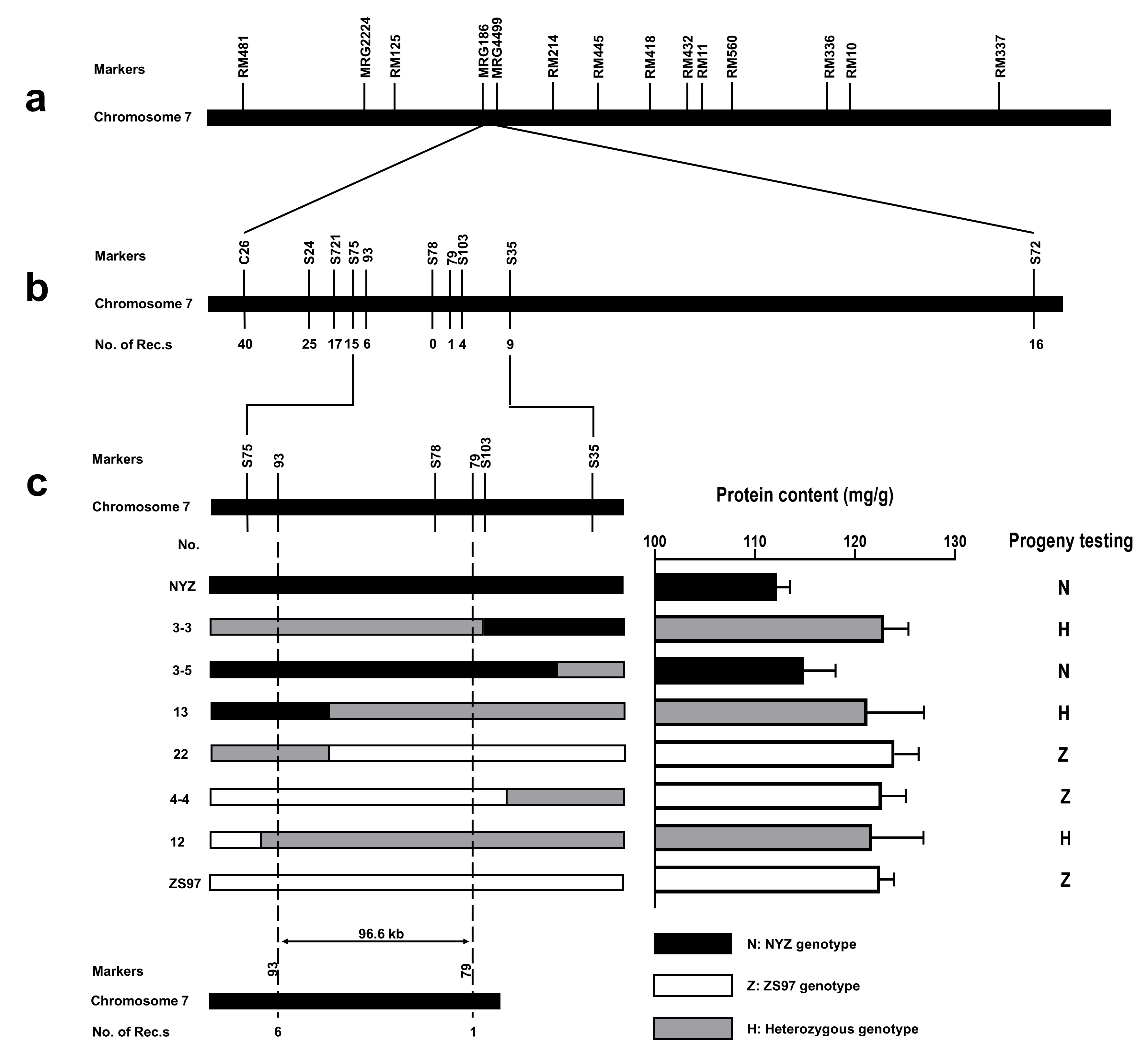


**Supplementary Figure 2 Map-based cloning of *qPC7*.** (**a**) Location of *qPC7* on the genetic linkage map of chromosome 7. (**b**) Recombinants in the *qPC7* interval. The number below each marker indicates the number of recombinants (Rec.) between *qPC7* and the molecular marker. (**c**) Genotypes and phenotypes of recombinants. The *qPC7* genotype of each recombinant was confirmed by progeny test.


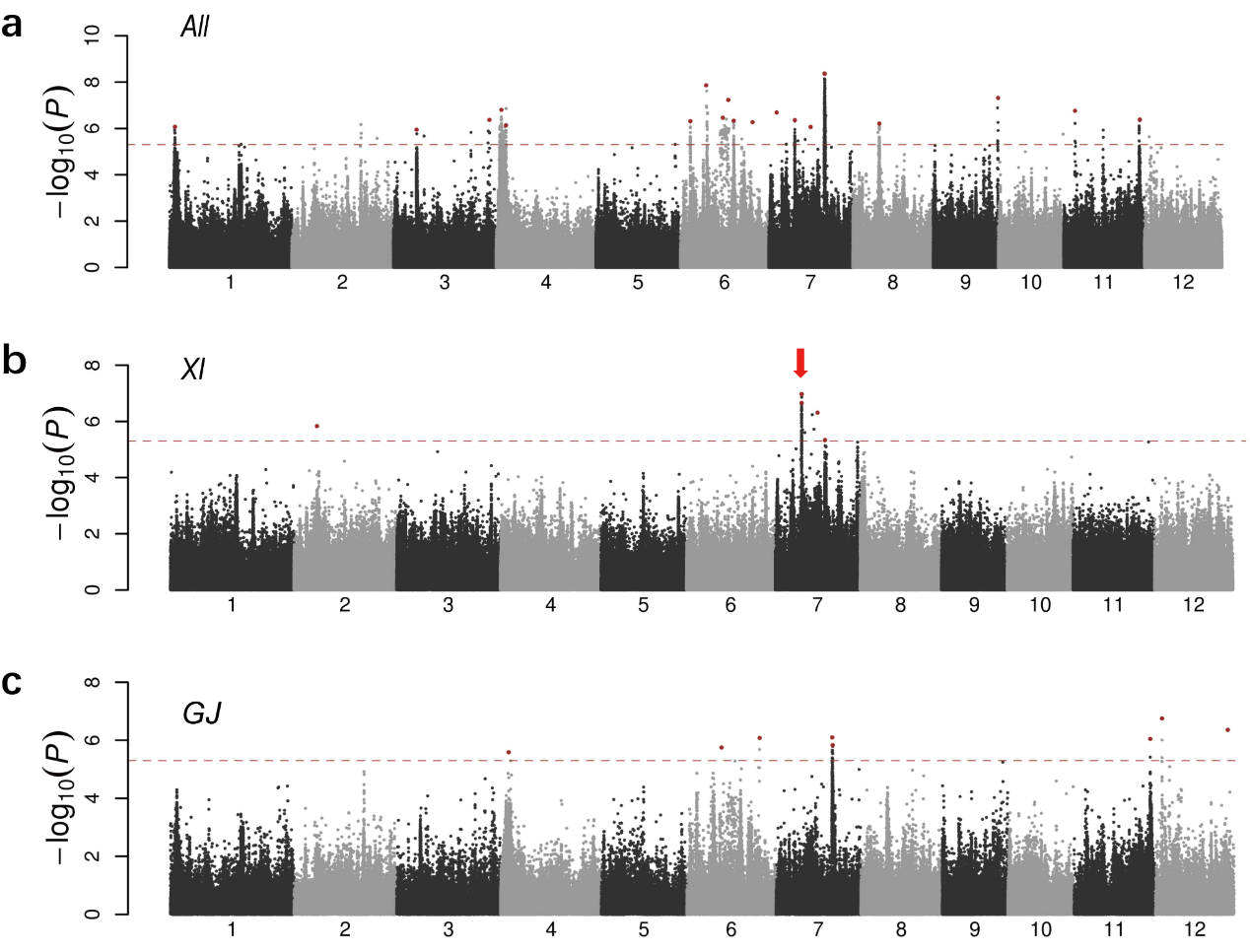


**Supplementary Figure 3 Manhattan plots depicting GWAS results for protein content of milled rice in 2015 using a linear-mixed model.** Red arrowheads indicate the position of *Ghd7*. All, total population; *XI*, *Xian*/Indica subpopulation; *GJ*, *Geng*/Japonica subpopulation.


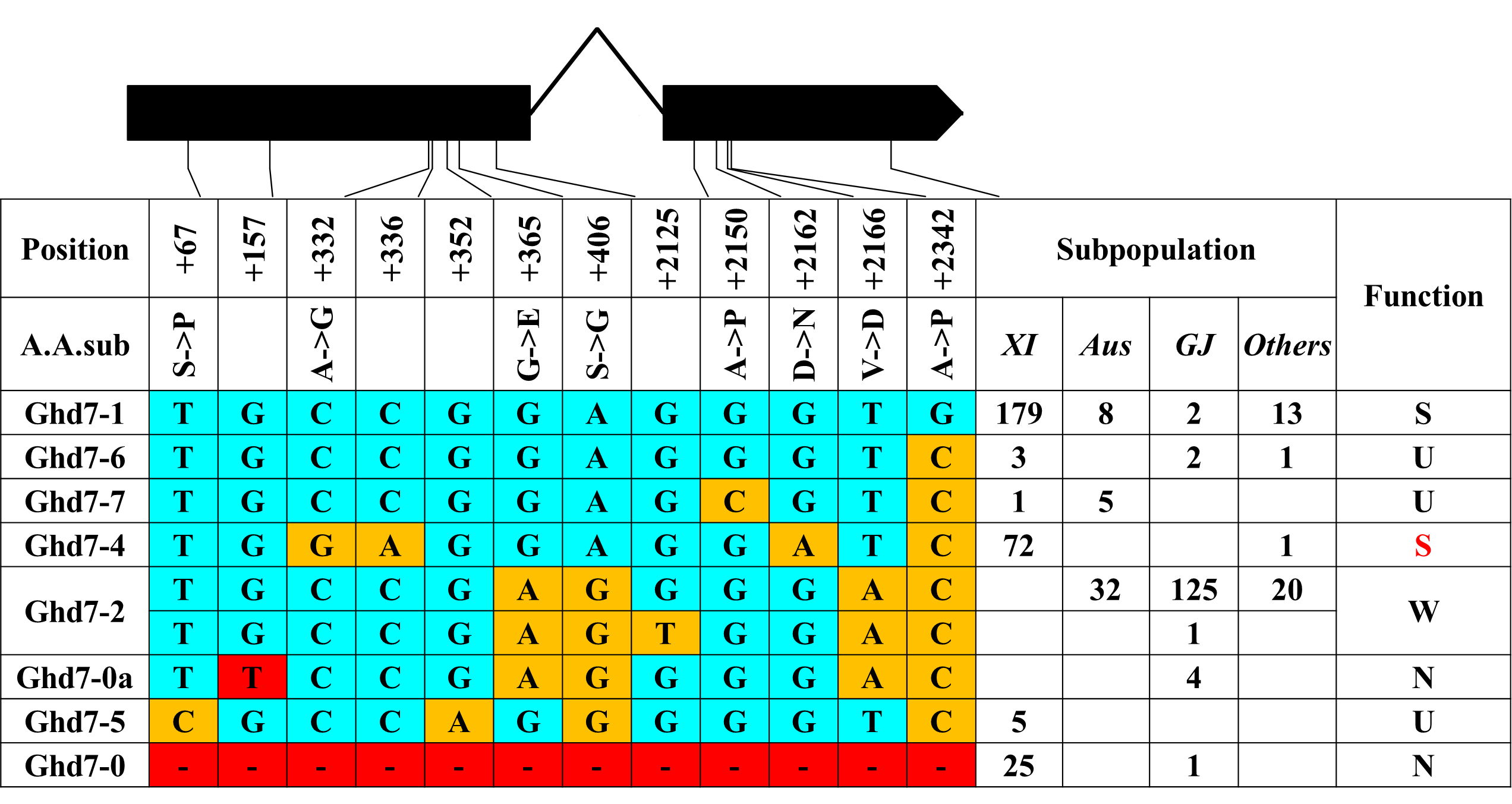


**Supplementary Figure 4 Natural variation analysis of *Ghd7* in a 533 rice accession panel.** Nucleotide polymorphisms in the *Ghd7* CDS region were identified with reference to the Minghui 63 genome sequence (Ghd7-1). Polymorphic nucleotides are marked in different colors. The frame-shift mutation (Ghd7-0a) generating a premature stop codon is marked in red. Ghd7-0 is a deletion of the entire gene (ZS97 type). Functions are described as S, strong; U, uncertain; W, weak; and N, non-functional. The black-filled polygons represent the coding region, and the peaked line between the polygons represents the intron.


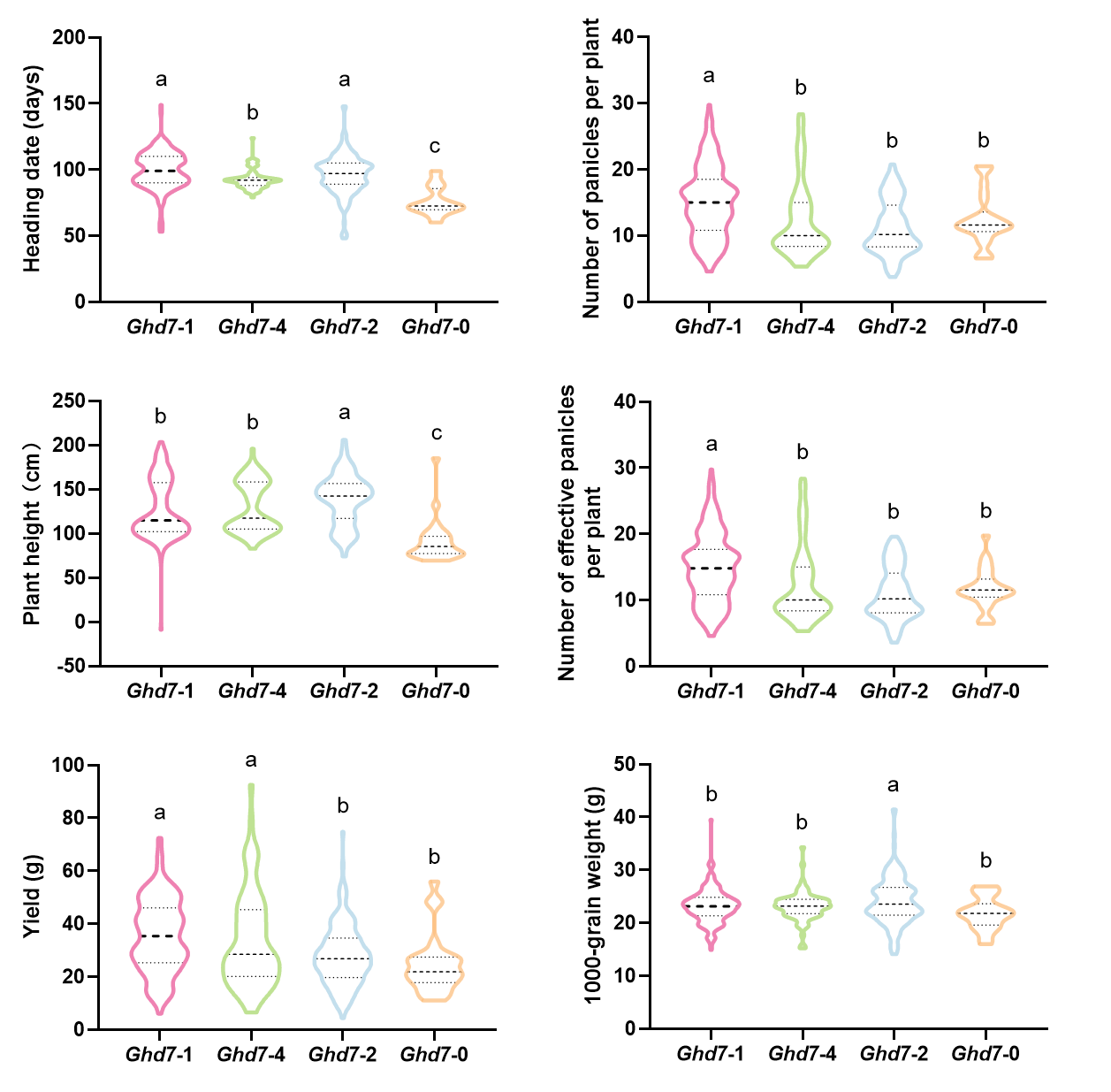


**Supplementary Figure 5 Analysis of agronomic traits of four major *Ghd7* haplotypes based on phenotypic data from 533 rice varieties.**  Data are means ± s.e.m (*n* ≥ 26); different letters represent significant differences (*P* <0.05, Duncan’s multiple range test).

**
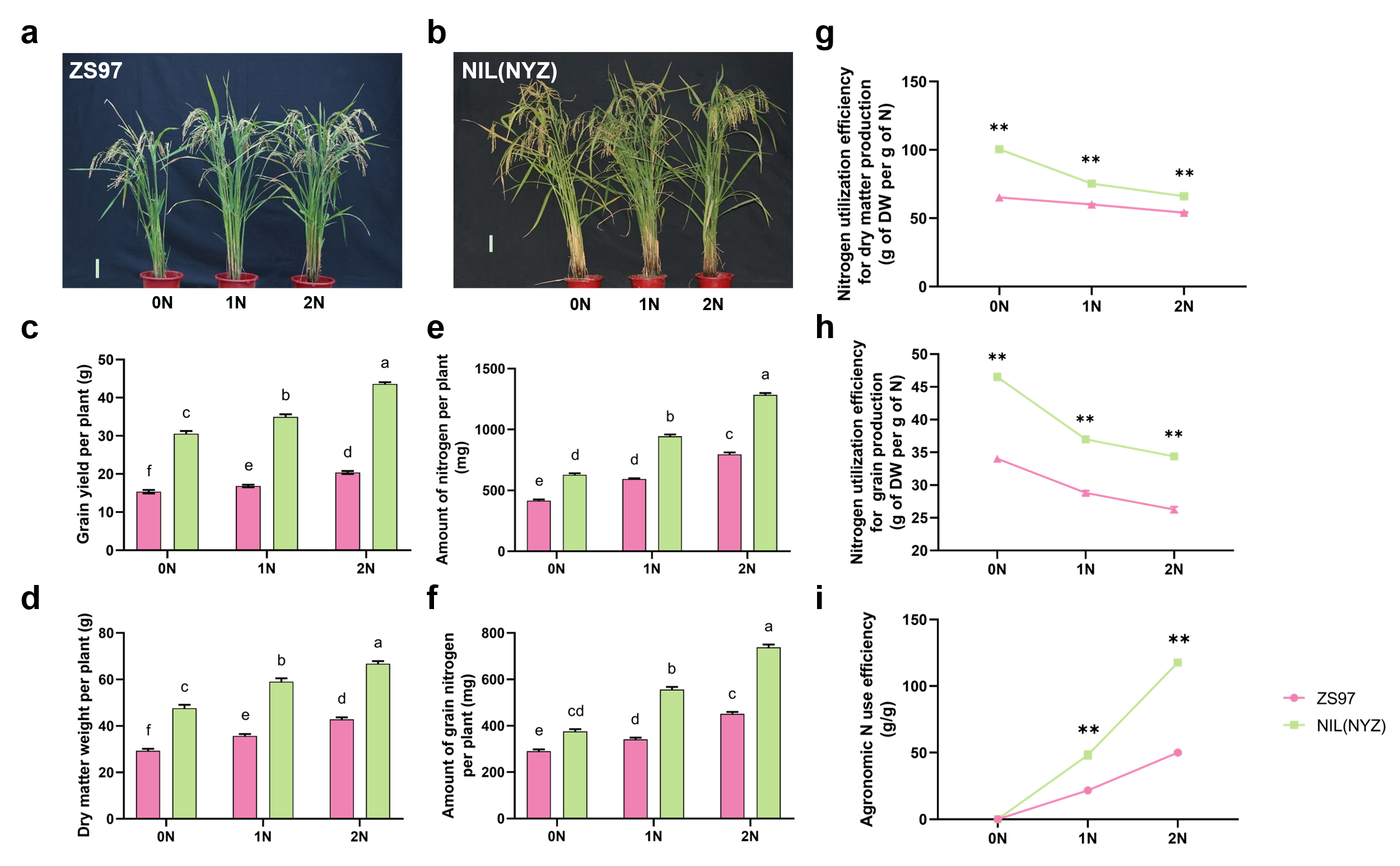
**

**Supplementary Figure 6 Effects of *Ghd7* on grain yield and NUE of rice in a field experiment.** (a-b) Appearance of two NILs under different nitrogen levels. Bar, 10 cm. (c) Grain yield per plant. (d) Dry matter weight per plant. (e) Amount of nitrogen per plant. (f) Amount of grain nitrogen per plant. (g) Nitrogen utilization efficiency for dry matter production and (h) Nitrogen utilization efficiency for grain production. (i) Agronomic nitrogen utilization efficiency for grain production In (c-f), data are means ± s.e.m (*n* ≥ 13); different letters represent significant differences (*P* <0.05, Duncan’s multiple range test). In (g-i), data are means ± s.e.m (*n* ≥ 13); ** indicate significant differences at *P* <0.01; *t*-tests.


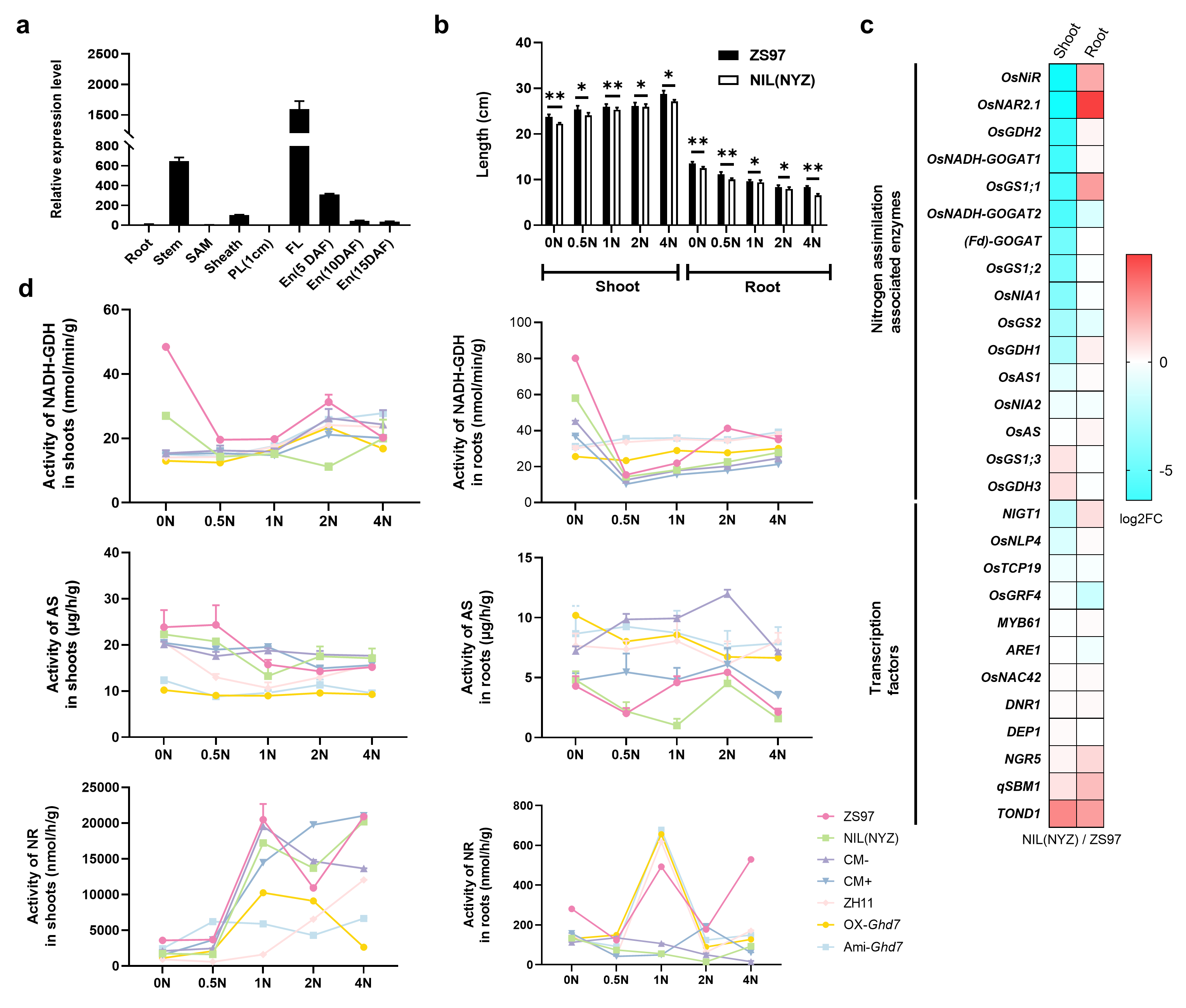


**Supplementary Figure 7 Ghd7 responds positively to nitrogen and strongly inhibits nitrogen assimilation.** (**a**) Expression levels of *Ghd7* in different tissues of NIL(NYZ). SAM, shoot apical meristem; PL, panicle; FL, flag leaf; En, endosperm; DAF, days after flowering. (**b**) Shoot and root lengths of NILs under different nitrogen concentrations. (**c**) Expression levels of nitrogen assimilation-related genes in the shoot and root of NILs at 15 days after germination. (**d**) Activities of NADH-glutamate dehydrogenase (NADH-GDH), asparagine synthase (AS) and nitrate reductase (NR) in the shoot and root of NIL seedlings. Data are means ± s.e.m. In **(b, d)**, *n* ≥ 9; in **(c)**, *n* = 3.


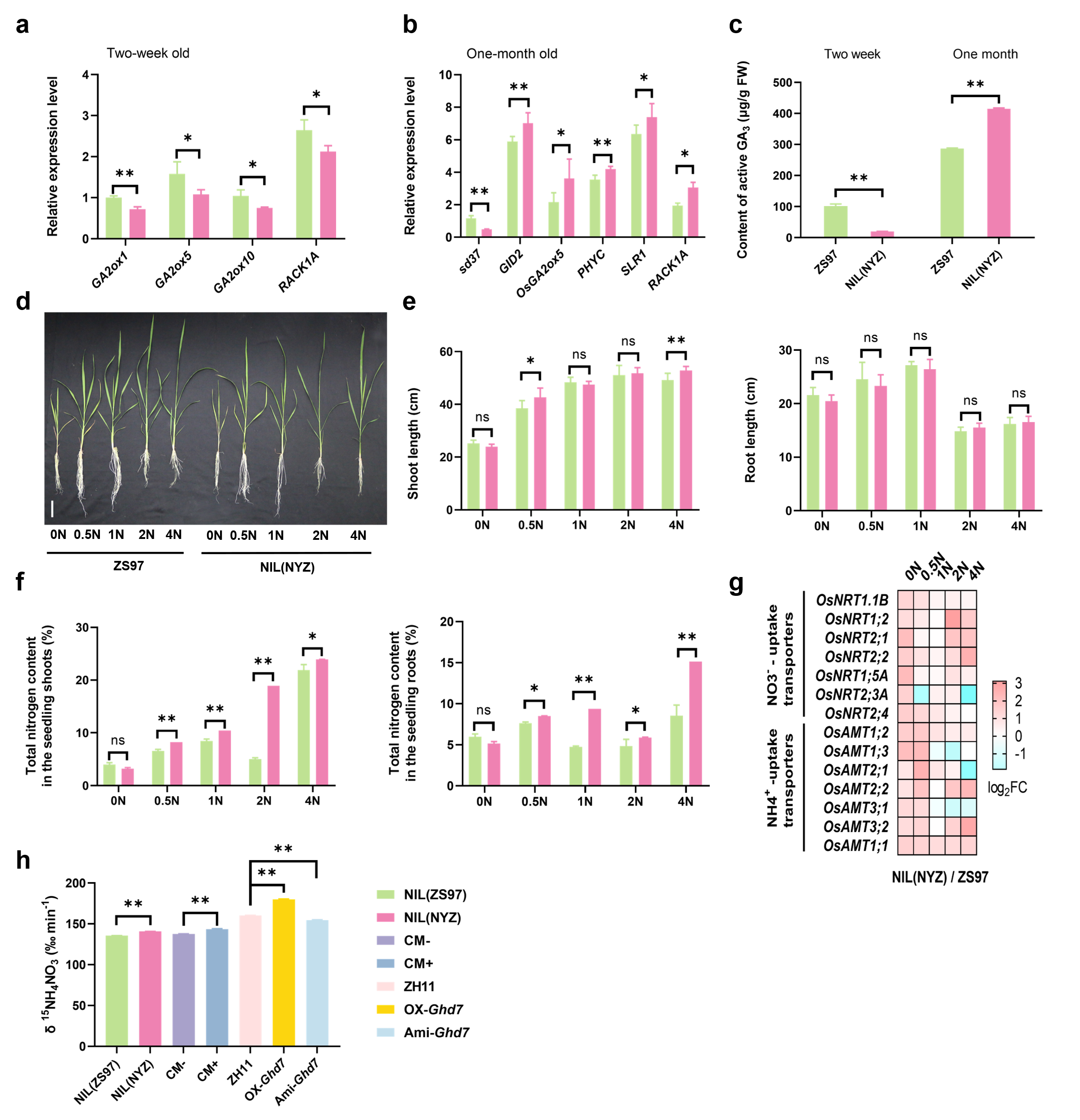


**Supplementary Figure 8 One-month nitrogen treatment experiment on NILs.** (**a**) Relative expression levels of 4 differentially expressed genes involved in GA synthesis and ABA accumulation in two-week old seedlings of NILs grown in the field under natural light conditions. Expression levels were determined by qRT-PCR using RNA samples from shoot. (**b**) Relative expression levels of 6 differentially expressed genes involved in GAs synthesis and ABA accumulation in one-month old seedlings of NILs grown in the field under natural light conditions. Expression levels were determined by qRT-PCR using RNA samples from shoot. (**c**) Endogenous active GA_3_ levels in two-week old and one-month old seedlings of NILs grown in the field under natural light conditions by HPLC analysis. (**d**) NIL seedlings subjected to different nitrogen conditions in hydroponic solution. Bar, 5 cm. (**e**) Shoot and root lengths of NILs grown in different nitrogen levels. (**f**) Total nitrogen contents of NILs grown under different nitrogen conditions. (**g**) Expression levels of 14 genes involved in nitrogen uptake and transport in NILs grown under different nitrogen conditions. Expression levels were determined by qRT-PCR using RNA samples from roots, *n* = 3. (h) Analysis of nitrogen uptake rate of one-month-old NILs and the *Ghd7*-related transgenic seedlings grown in the presence of 1.46 mM NH_4_NO_3_. Data are mean values of three biological replicates with s.e.m (*n* = 6 seedlings). * and **, significantly different at *P* <0.05 and *P*<0.01, respectively, *t*-tests. "ns" indicates no significant difference.


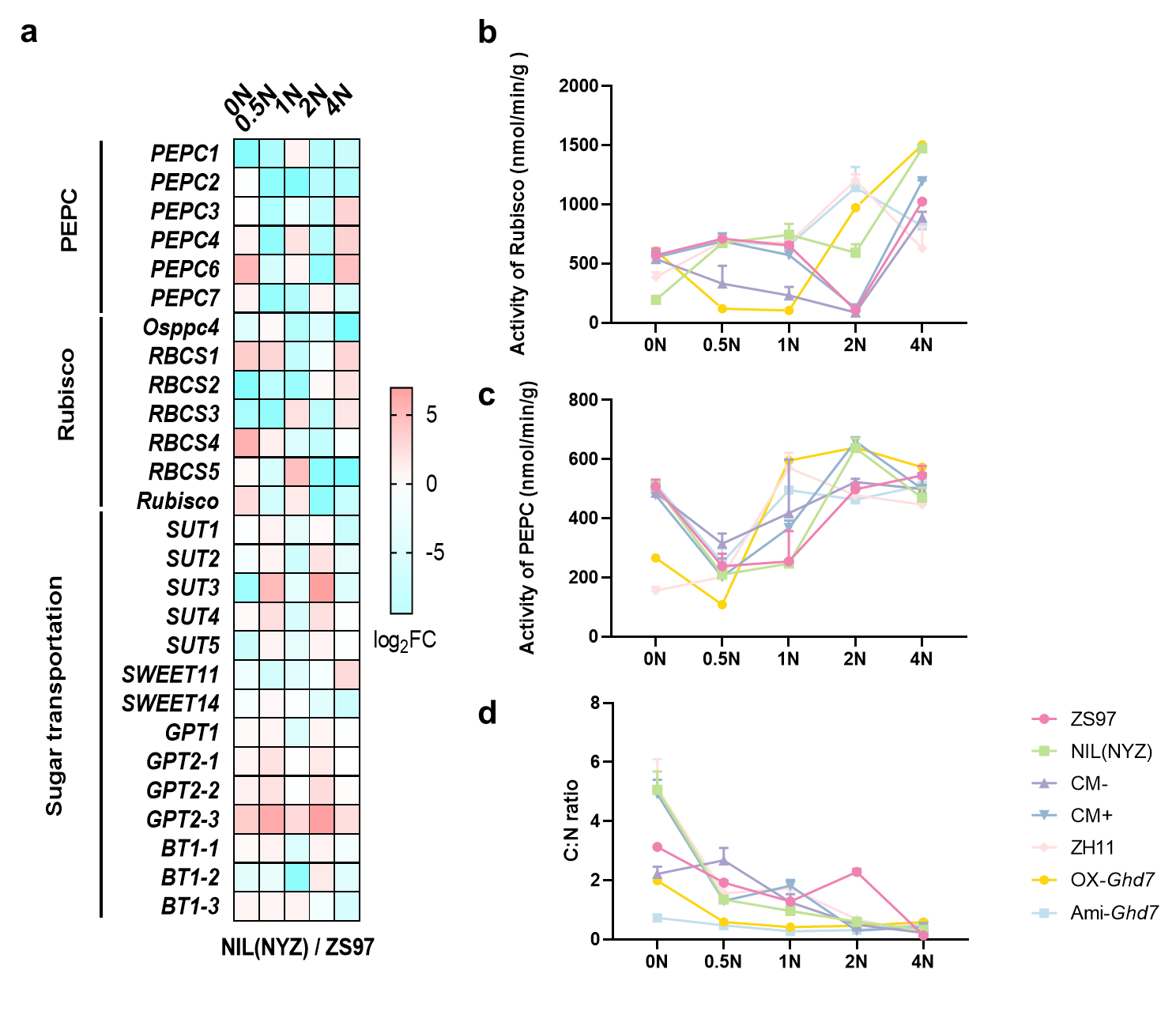


**Supplementary Figure 9 Effect of *Ghd7* on carbon metabolism. (a)** Relative expression levels of phosphoenolpyruvate carboxylase (PEPC) family members, Rubisco (Ribulose bisphosphate carboxylase oxygenase) family members and sugar transportation related genes. **(b, c)** Activities of ribulose-1,5-diphosphate carboxylase (Rubisco) and phosphoenolpyruvate carboxylase (PEPC) in NILs. (**d**) C:N ratios in one-month-old NIL seedlings. Data are means ± s.e.m (*n* = 3). * and **, significantly different at *P* <0.05, and *P* <0.01, rrespectively; t-tests.


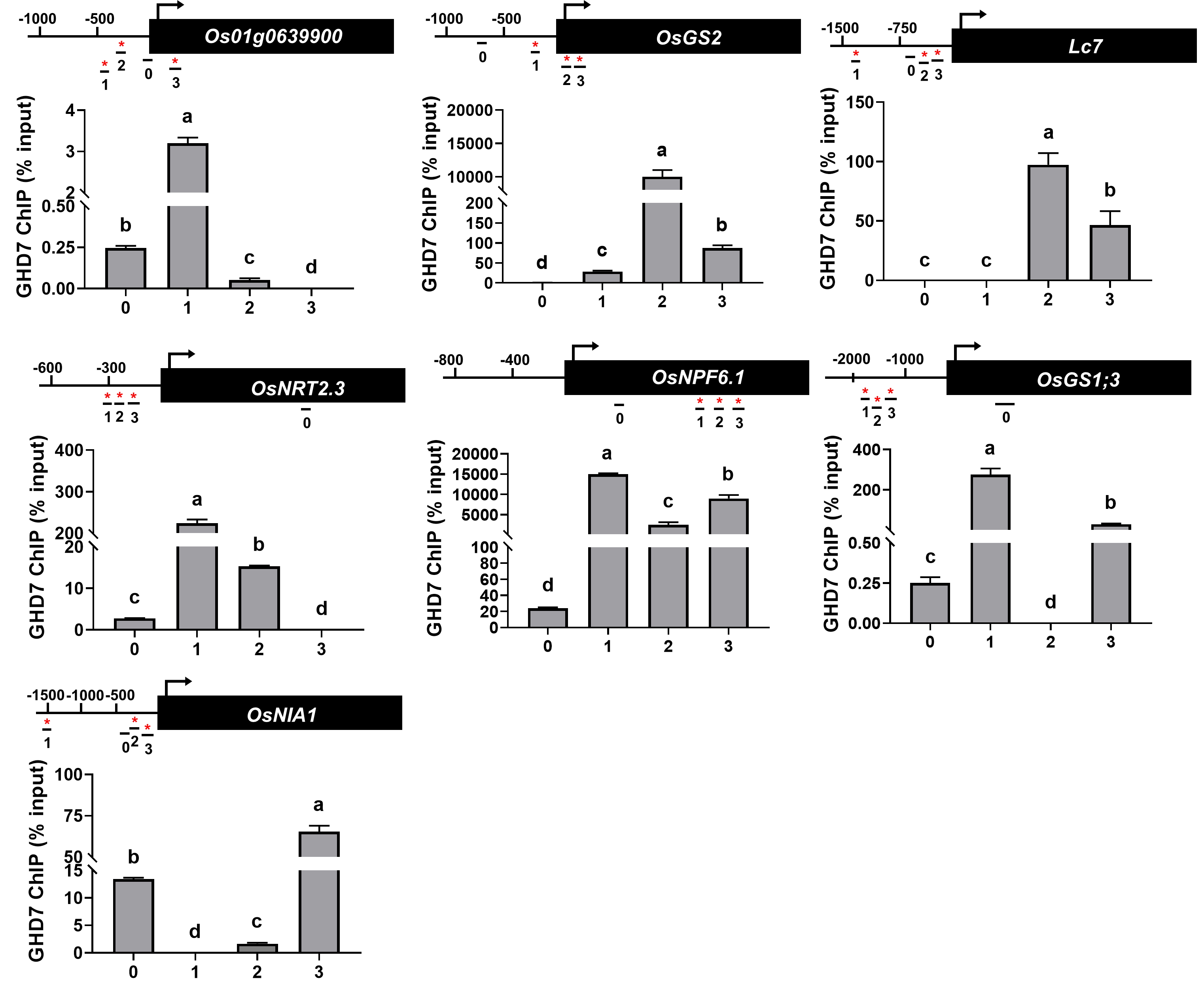


**Supplementary Figure 10 Analysis of the DNA-binding capability of GHD7 protein by ChIP.** GHD7 mediated ChIP–qPCR enrichment (relative to input) of CCACC-containing fragments (marked with a red asterisk) from *Os01g0639900*, *OsGS2*, *Lc7*, *OsNRT2.3*, *OsNPF6.1*, *OsGS1;3* and *OsNIA1*. DNA fragments not marked with an asterisk and numbered 0 do not contain CCACC motif. For genes containing more than 3 CCACC motifs, three were randomly selected for enrichment analysis. Different letters denote significant differences (*P* <0.05) from a Duncan’s multiple range test.


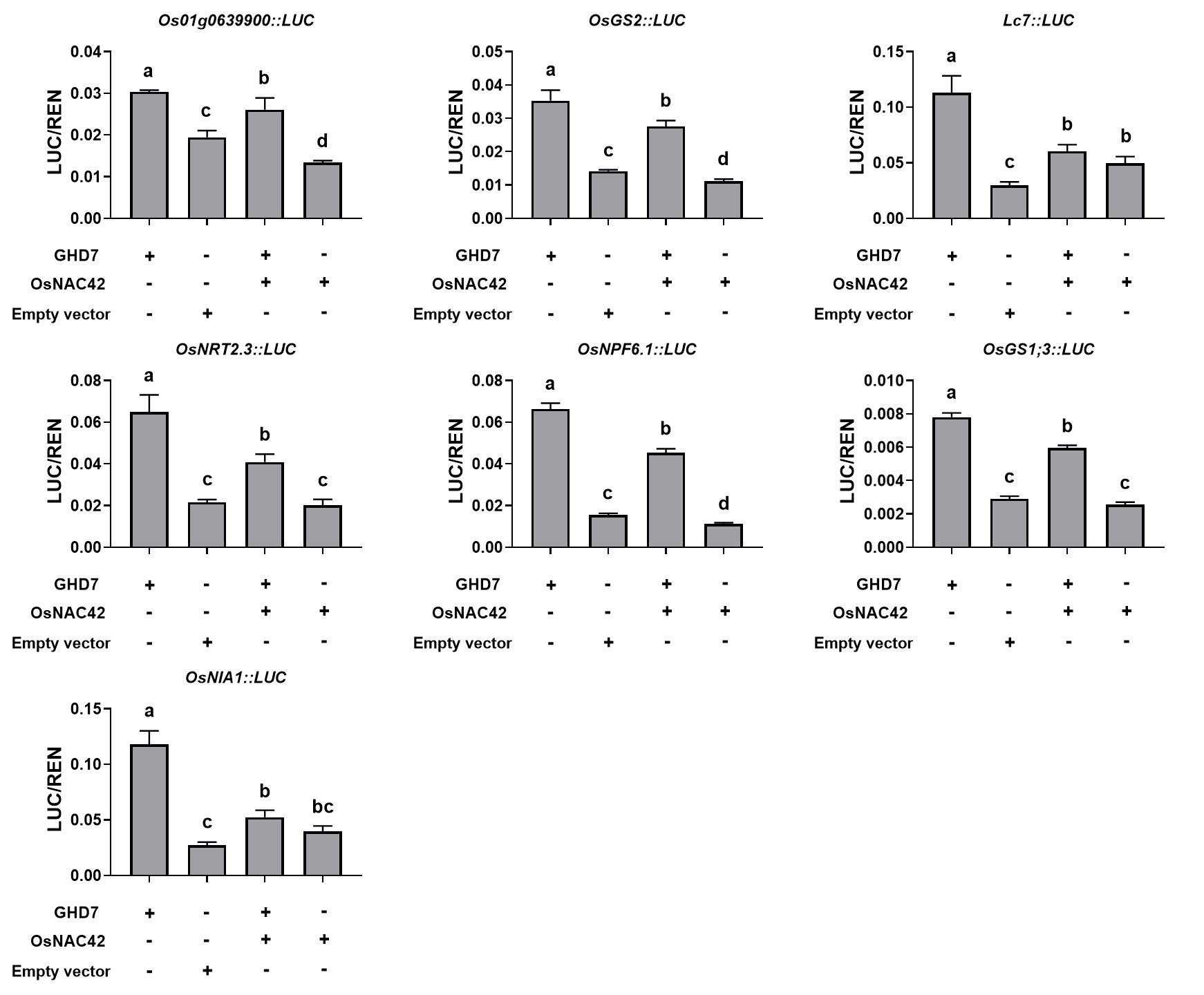


**Supplementary Figure 11 Transcriptional activity analysis in rice protoplasts** **Data presented are the mean values of 10 technical replicates with s.e.m.** Different letters denote significant differences (*P* <0.05) from a Duncan’s multiple range test.


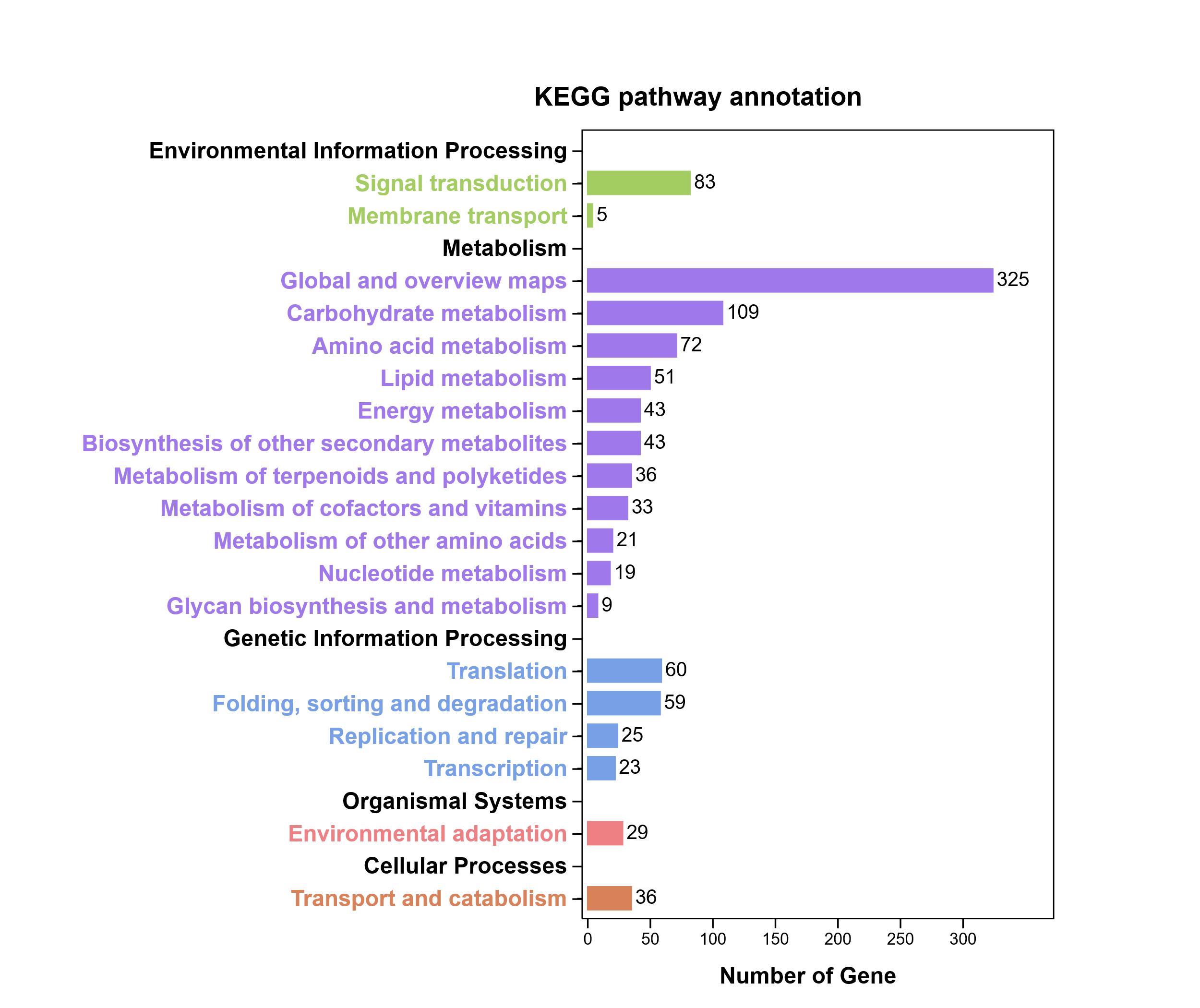


**Supplementary Figure 12 Kyoto Encyclopedia of Genes and Genomes (KEGG) analysis of downstream potential target genes regulated by Ghd7 in endosperm.**


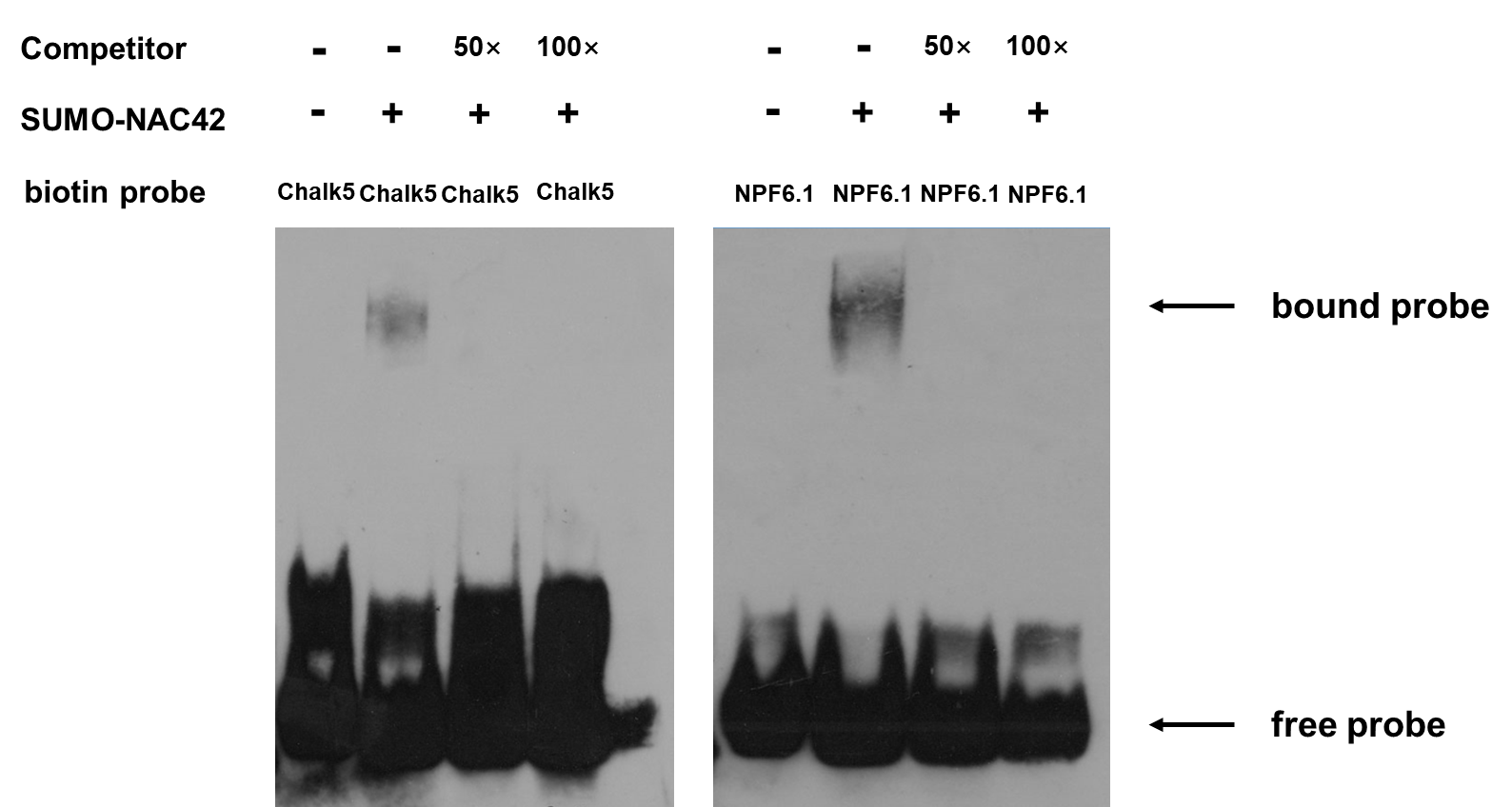


**Supplementary Figure 13 EMSA of in vitro OsNAC42 binding to specific motifs in the promoters of *Chalk5* and *OsNPF6.1*.** Unlabeled probes were used for competition.

**
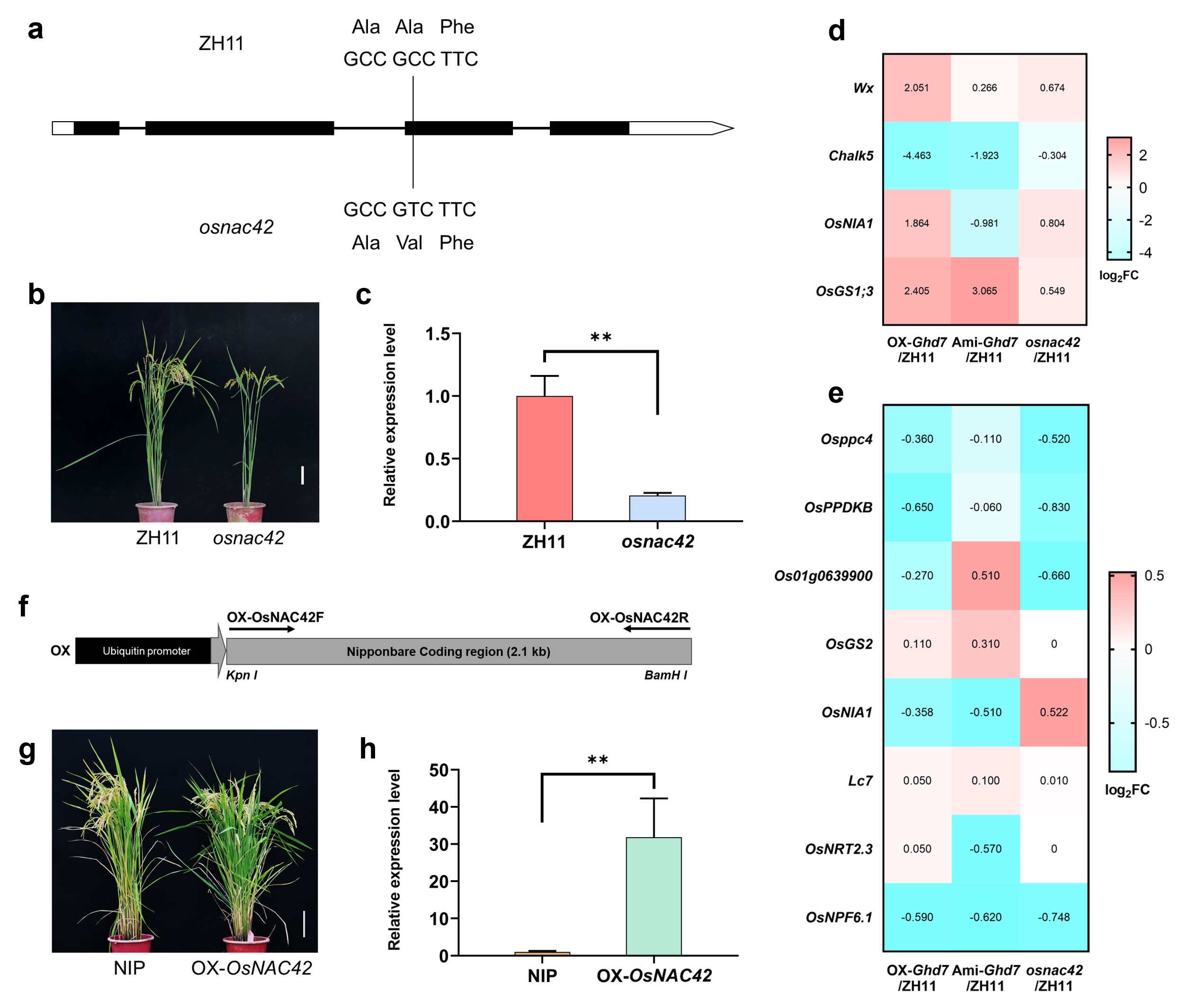
**

**Supplementary Figure 14 Functional analysis of *OsNAC42*.** (**a**) The sequence comparison between *OsNAC42* tilling mutant (*osnac42*) and ZH11 at the mutation site. (**b**) Plant architecture of ZH11 and *osnac42* mutant. Bar, 10 cm. (**c**) Relative expression levels of *OsNAC42* in ZH11 and *osnac42* mutant. Expression heatmap of target genes involved in carbon and nitrogen metabolism of Ghd7-OsNAC42 complex in endosperm (**d**) and seedlings (**e**). (**f**) Schematic of vectors for transgenic analysis. The *OsNAC42* coding region of Nipponbare was inserted into the vector pU2301-flag under control of the CAMV35S promoter to prepare the overexpression construct (OX-*OsNAC42*). Arrows represent the direction of PCR primers. (**g**) Plant architecture of NIP and OX-*OsNAC42*. Bar, 10 cm. (**h**) Relative expression levels of *OsNAC42* in NIP and OX-*OsNAC42*. In (**c**) and (**h**), leaf samples were used for total RNA extraction, and three biological replicates were used for qRT-PCR. Data are means ± s.e.m. **, significantly different at *P* <0.01; *t*-tests.


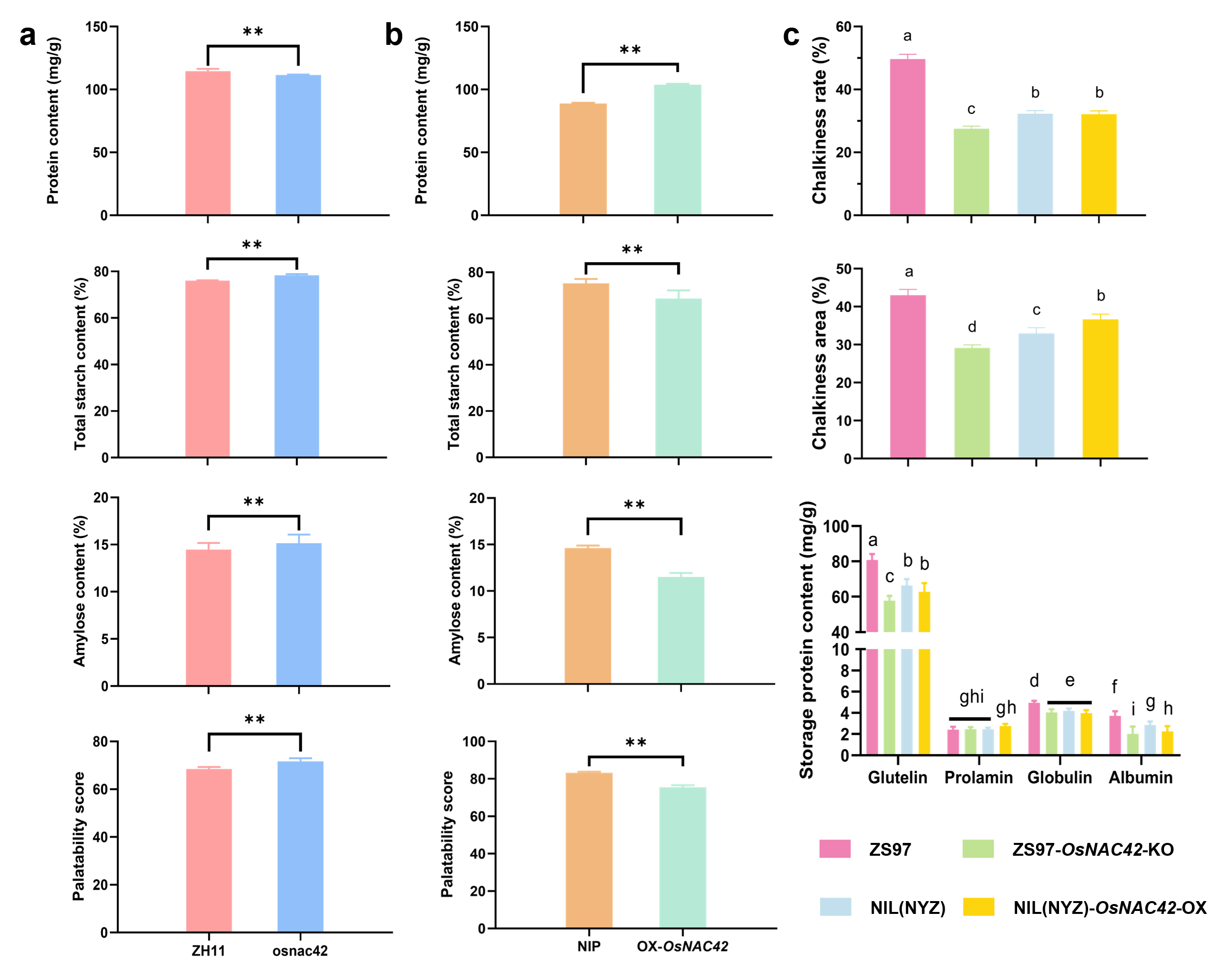


**Supplementary Figure 15 Quality phenotypes of *OsNAC42* related genetic materials.** (**a**) Main grain quality traits in *OsNAC42* tilling mutant (*osnac42*) and ZH11, including grain protein content, total starch content in grain, amylose content in grain and palatability score. (**b**) Main grain quality traits in *OsNAC42*-overexpressing plants (OX-*OsNAC42*) and Nipponbare (NIP), including grain protein content, total starch content in grain, amylose content in grain and palatability score. (**c**) Chalkiness and grain storage protein contents in ZS97, NIL(NYZ), ZS97-*OsNAC42*-KO and NIL(NYZ)-*OsNAC42*-OX plants. In (a,-c), data are means ± s.e.m (*n* = 20). ** indicate significant differences at *P* <0.01; t-tests. Different letters denote significant differences (*P* <0.05) from a Duncan’s multiple range test.


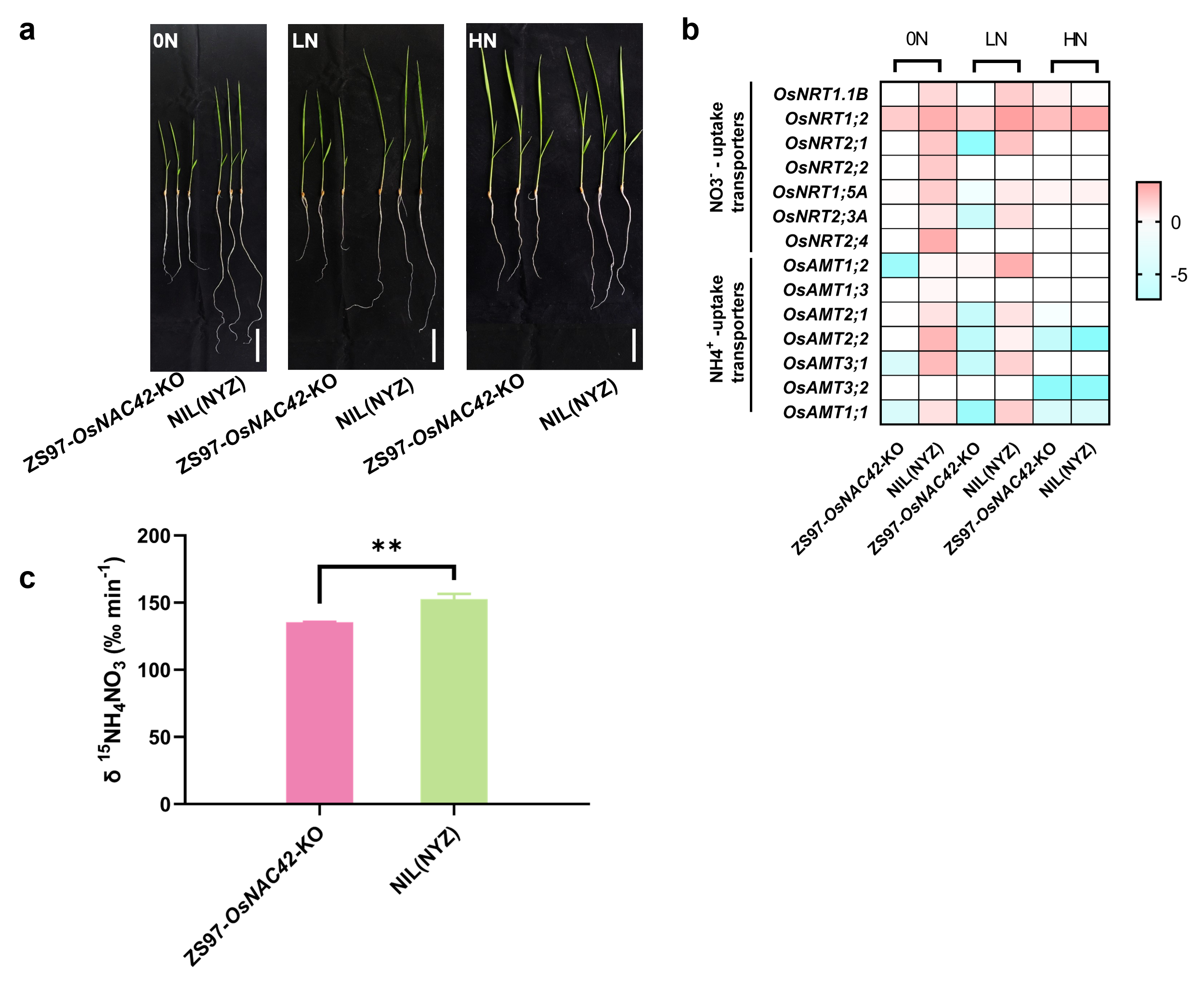


**Supplementary Figure 16 The response of rice to nitrogen depends on the Ghd7-OsNAC42 function module.** (**a**) One-week nitrogen treatment experiment on the seedlings of ZS97-OsNAC42-KO and NIL(NYZ). Bar, 5 cm. (**b**) Expression levels of 14 genes involved in nitrogen uptake and transport in ZS97-*OsNAC42*-KO and NIL(NYZ) grown under different nitrogen conditions. Expression levels were determined by qRT-PCR using RNA samples from roots, *n* = 3. (c) Analysis of nitrogen uptake rate of one-week-old ZS97-*OsNAC42*-KO and NIL(NYZ) seedlings grown in the presence of 1.46 mM NH_4_NO_3_. Data are mean values of three biological replicates with s.e.m (*n* = 6 seedlings). **, significantly different at *P*<0.01, *t*-tests.


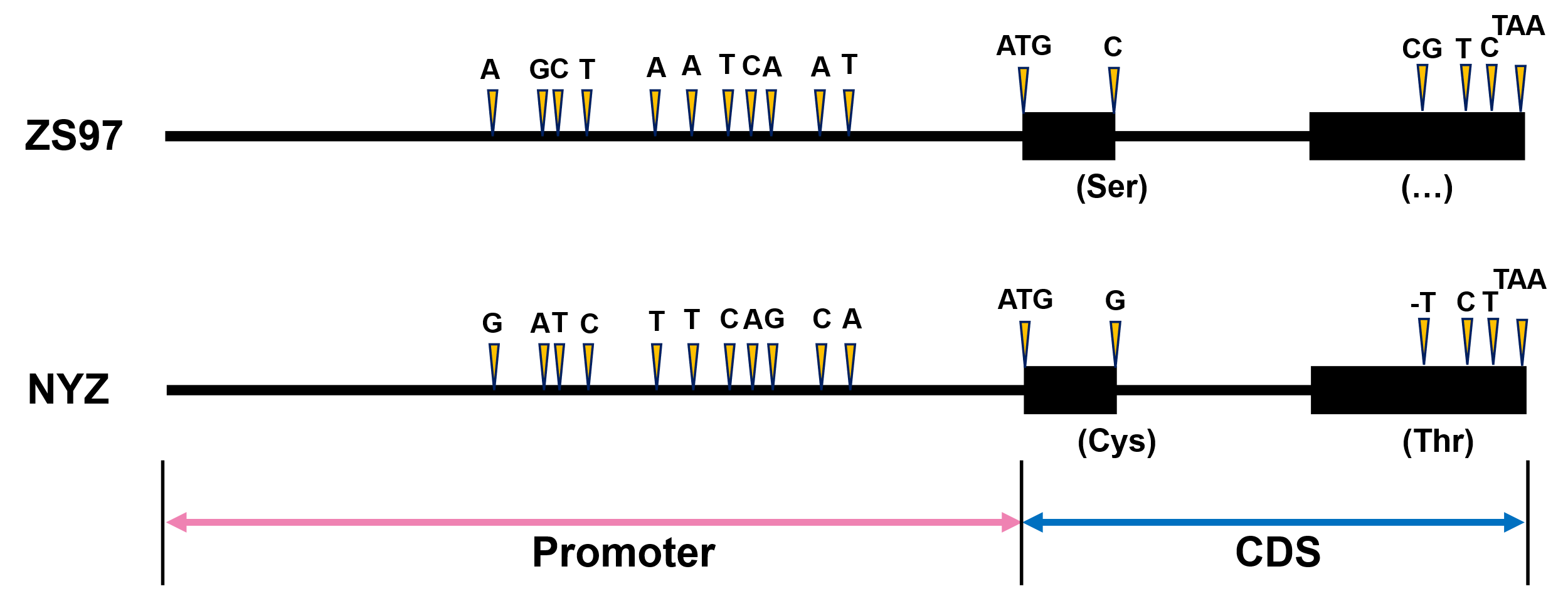


**Supplementary Figure 17 DNA sequence polymorphisms in *LOC_Os07g15820* from ZS97 and NYZ.** SNPs between the two alleles were marked by inverted triangles with bases above. ‘-’ represents deletion of a base. Exons are indicated by black boxes in the CDS region and the black lines represent the promoter or the intron. Amino acids in brackets represent variations in the two proteins.


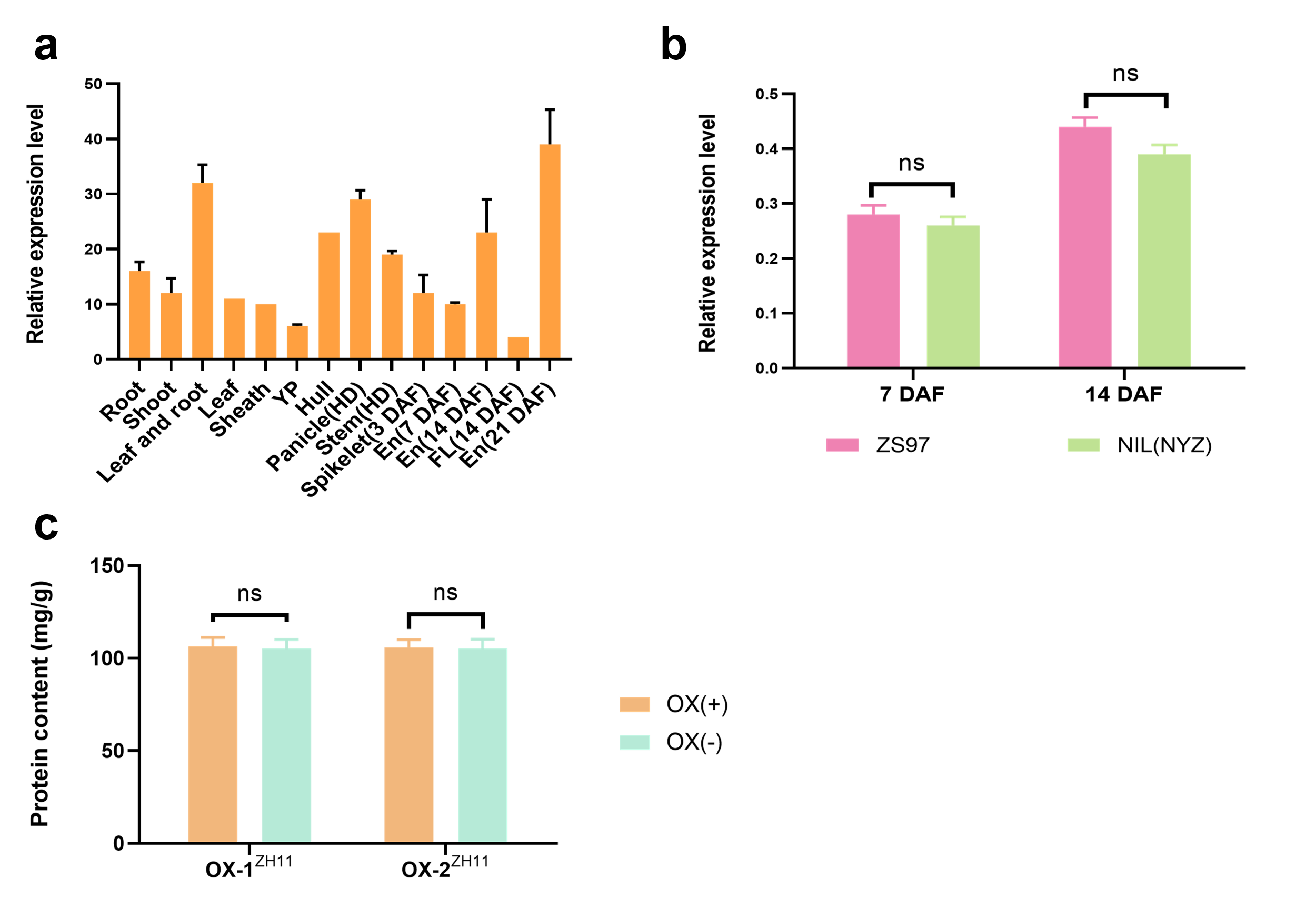


**Supplementary Figure 18 Expression pattern of *LOC_Os07g15820* and grain protein content of *LOC_Os07g15820* T_1_ transgenic plants.** (**a**) Expression levels of *LOC_Os07g15820* in various tissues of ZS97B based on microarray data^1^. (**b**) Comparative expression patterns of *LOC_Os07g15820* alleles from ZS97 and NIL(NYZ) in endosperm at 7 and 14 days after flowering. Expression levels were determined by qRT-PCR using RNA samples from endosperms with three biological replications. Data are means ± s.e.m (*n* = 3). (**c**) Grain protein contents of *LOC_Os07g15820* T_1_ transgenic plants. OX-1^ZH11^ and OX-2^ZH11^ are transgenic ZH11 plants carrying p*35S*::c*LOC_Os07g15820*^NYZ^ and p*35S*::c*LOC_Os07g15820*^ZS97^ constructs, respectively. OX(+) and OX(-) are transgenic positive and negative plants, respectively. Data are mean ± SE (*n* = 15). "ns" indicates no significant difference.


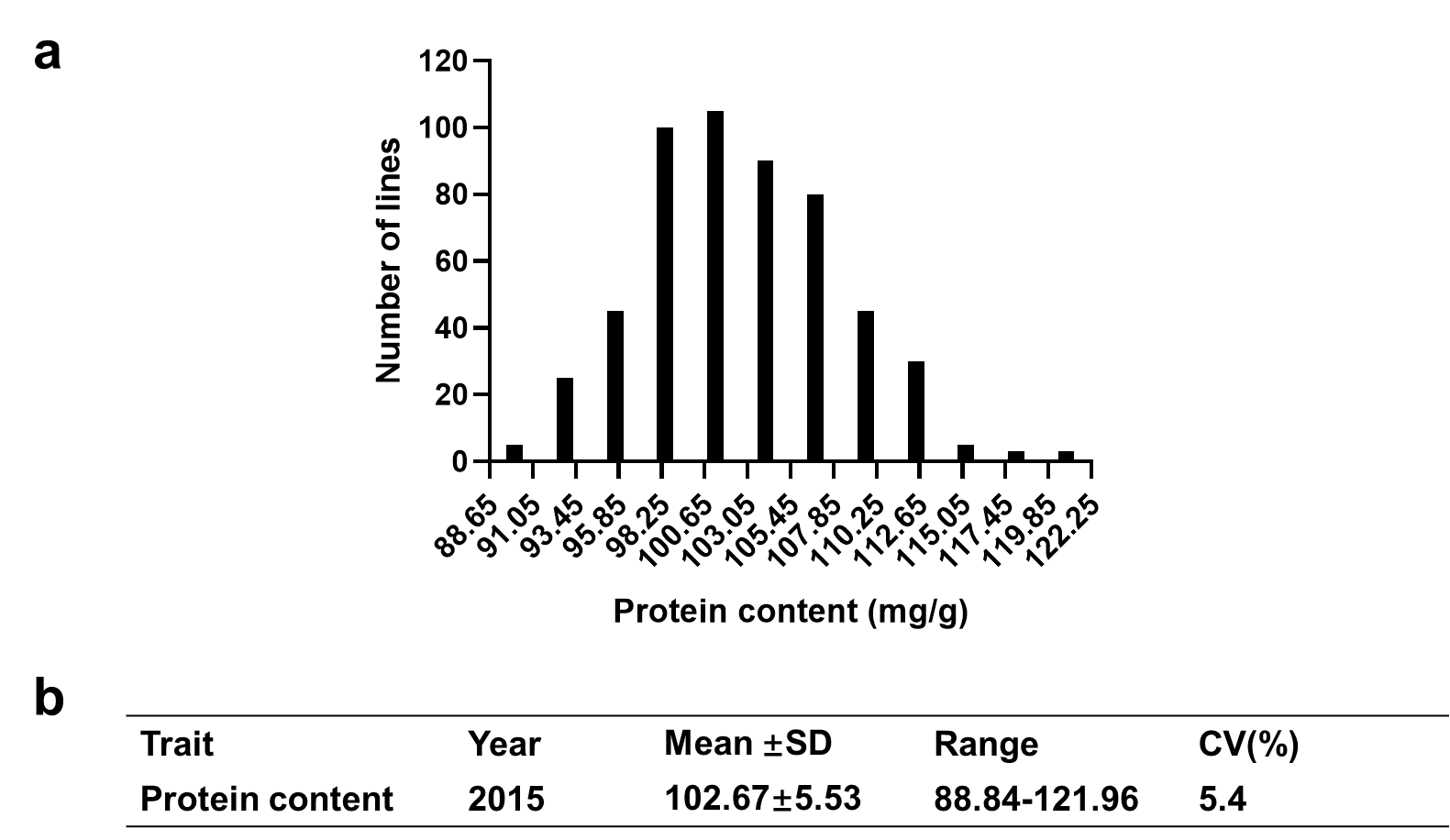


**Supplementary Figure 19 Distribution of protein contents in milled rice from a panel 533 germplasm accessions grown in 2015.** (**a**) Frequency distribution; (**b**) Mean and range of protein contents.

1 Wang, L. *et al.* A dynamic gene expression atlas covering the entire life cycle of rice. *Plant J* **61**, 752-766, doi:10.1111/j.1365-313X.2009.04100.x (2010).
